# Improving crowdsourcing for AI through cognitive-inspired data engineering

**DOI:** 10.3758/s13428-026-03160-4

**Published:** 2026-09-01

**Authors:** Gunnar P. Epping, Andrew Caplin, Erik Duhaime, William R. Holmes, Daniel Martin, Jennifer S. Trueblood

**Affiliations:** 1 Cognitive Science Program, Indiana University, Indiana, USA; 2 Department of Psychological and Brain Sciences, Indiana University, Indiana, USA; 3 Centaur Labs, Massachusetts, USA; 4 Department of Economics, New York University, New York, USA; 5 Department of Mathematics, Indiana University, Indiana, USA; 6 Department of Economics, University of California, Santa Barbara, USA

**Keywords:** Crowdsourcing, Subjective probability judgments, Recalibration, Cognitive biases, Medical image classification, Machine learning training data

## Abstract

Crowdsourcing offers a fast and cost-efficient approach to obtaining human-labeled datasets. However, crowdsourced datasets and the models trained on them can inherit the cognitive constraints and biases of their annotators. In a process we refer to as cognitive-inspired data engineering, we investigate whether ideas from cognitive science can be applied to mitigate the presence of cognitive constraints and cognitive biases in crowdsourced datasets and, as a result, improve the performance of models trained on these datasets. We evaluate our approach by crowdsourcing labels for medical image diagnostic tasks using two different crowdsourcing platforms across two experiments. In Experiment 1, we collect subjective probability judgments from novice annotators through Amazon Mechanical Turk and, in Experiment 2, we collect subjective probability judgments and binary classifications from skilled annotators through DiagnosUs, a crowdsourcing platform specializing in medical and scientific data annotation. In both experiments, we find that recalibrating subjective probability judgments reduces bias, yielding more accurate crowdsourced datasets and more accurate models trained on them. Our results suggest that cognitive-inspired data engineering offers a promising avenue to improve the quality of crowdsourced datasets, with consistent downstream benefits for machine learning models.

**Supplementary Information:**

The online version contains supplementary material available at https://doi.org/10.3758/s13428-026-03160-4.

## Introduction

Integrating artificial intelligence (AI) systems into a wide range of perceptual decision-making tasks, such as airport baggage screening decisions (Vukadinovic & Anderson, 2022), refereeing decisions in sporting events (Thomas-Acaro & Meneses-Claudio, 2024), medical image decisions (Panayides et al., 2020), and threat detection based on satellite and sonar images (Surma, 2024), holds great promise. However, in many cases, the training of these systems relies on humans annotating images. Despite the recent success of unsupervised machine learning (ML) (Van Gansbeke et al., 2020), training on human-labeled data is still the norm and is a multi-billion-dollar business. For example, Scale AI was valued at $13.8 billion in May 2024 (Wang, 2024).

Having a single person annotate an entire dataset can be very time-consuming, if not impossible, since many ML models require thousands, if not millions, of training instances to reach high levels of accuracy on out-of-sample data. As a result, online crowdsourcing (Deng et al., 2009) has emerged as a primary avenue to facilitate the curation of human-labeled datasets. It has become increasingly popular with the availability of platforms, such as Amazon Mechanical Turk (MTurk) (Chen et al., 2011), which connect ML practitioners with large pools of annotators (Eickhoff et al., 2012). Crowdsourcing dramatically reduces the amount of time required to collect human labels by breaking down the task of annotating an entire dataset into several micro-tasks where people only annotate a small subset of the data.

High-skill domains such as medical image diagnostics initially shied away from online crowdsourcing because many tasks are complex and require some level of expertise to accurately annotate data (Tucker et al., 2019). Hiring individuals with domain-specific expertise (experts) adds another hurdle to creating labeled datasets because experts are in short supply and more expensive than individuals on crowdsourcing platforms. The need for expert annotators has led to a lack of large labeled datasets in high-skill domains, and this lack is the primary bottleneck in developing AI systems in these domains (Ørting et al., 2019; Willemink et al., 2020). However, the belief that only experts can accurately annotate these datasets is, for the most part, unfounded. For example, while no one would expect an MTurk worker to diagnose images of potentially cancerous skin lesions as accurately as a board-certified dermatologist, the MTurk worker does not need to match the accuracy of the expert in order for the accuracy of the crowdsourced dataset to match that of the expert-labeled dataset. Crowdsourcing enables ML practitioners to collect several judgments on each image and harness the “Wisdom of the Crowds” (WoC). WoC describes a phenomenon where the consensus opinion of a group of people is more accurate compared to the single opinion of an individual within the same group (Galton, 1907; Surowiecki, 2005). The WoC is theorized to rely on functional diversity which, in the context of a classification task, describes the unique way people represent the task and arrive at their classification decision (Davis-Stober et al., 2014; Hong & Page, 2004). By aggregating annotations across a group of functionally diverse individuals, crowdsourcing leads to improved accuracy by identifying the common signal while negating noise among the annotators. Researchers have demonstrated that with either a skilled group of annotators or sufficiently sophisticated aggregation algorithms (algorithms used to combine several opinions into a single consensus label), crowdsourced labels can not only match but also exceed the accuracy of individual, credentialed experts in medical image diagnostics (Duhaime et al., 2023; Hasan et al., 2024; Maier-Hein et al., 2014). Therefore, crowdsourcing offers an antidote to the primary bottleneck in the development of AI systems in high-skills domains by reducing the cost and amount of time required to annotate the dataset without sacrificing accuracy compared to datasets annotated by experts.

Crowdsourcing does not solve all of the challenges involved in collecting human-annotated datasets, as both novices and experts alike have cognitive constraints and biases that influence their decision-making processes (Dror et al., 2018). Labeled datasets can inherit annotator biases, which then become embedded in models trained on that data (Bender et al., 2021; Bolukbasi et al., 2016). Since these biases enter the ML model development pipeline at its inception, they can be corrected by (1) de-biasing the data itself (Feldman et al., 2015; Jiang & Nachum, 2020; Kamiran & Calders, 2009), (2) by adapting the model algorithm to account for biases, such as correcting the model output post-training (Doherty et al., 2012; Hardt et al., 2016), or (3) by modifying the objective function (Goh et al., 2016; Kamishima et al., 2012; Zafar et al., 2017). We approach the problem by de-biasing the data itself. In a process we will refer to as “cognitive-inspired data engineering”, we investigate whether ideas from cognitive science can be applied to mitigate the presence of cognitive biases in crowdsourced datasets and, as a result, improve the performance of ML models trained on that data.

By “cognitive-inspired”, we refer to a conceptual approach where we use empirical findings and models from studies of human cognition (e.g., systematic judgment biases) to guide how crowdsourced judgments are collected, weighted, and corrected. This work introduces that perspective and demonstrates one instantiation through recalibration of crowdsourced judgments. Importantly, our approach does not involve joint optimization of human and machine decision processes, nor does it modify model architectures or training objectives. Instead, we intervene upstream by correcting systematic biases in annotator-level judgments, allowing both dataset quality and downstream model performance to improve as a consequence of improved data.

Two primary steps in the crowdsourcing process involve eliciting the annotations from individuals and aggregating annotations across individuals to create crowdsourced labels. For classification tasks, the most common method of eliciting annotations from human raters is having each rater provide single labels (i.e., selecting a single discrete class out of all possible classes) (Collins et al., 2022). While effective, single labels are inefficient because they constrain people to a small number of choice alternatives, potentially throwing away valuable information when annotators are unsure of the correct classification.

Subjective probability judgments, unlike single labels, allow people to express uncertainty in their decision. However, the additional information conveyed in these judgments is a double-edged sword. With the additional information, there are more ways for cognitive biases to enter the data. For example, with single labels, the main bias is a response bias, where a rater tends to provide one label more than the others. A response bias can arise due to a variety of factors, such as unbalanced base rates across classes (Roy & Lerch, 1996; Trueblood et al., 2021; Wolfe & Van Wert, 2010). In probability judgments, a response bias can appear with a rater favoring one side of the probability scale compared to the other. Another bias that appears in subjective probability judgments is over/underconfidence (Harvey, 1997; Keren, 1997). Similar to response biases, over/underconfidence can be caused by several factors such as the hard-easy effect (Suantak et al., 1996), where people are overconfident in difficult classification tasks and underconfident in easier ones.

As a result of response biases and over/underconfidence, subjective probability judgments rarely equate to the likelihood of being correct and are therefore poorly calibrated (Griffin & Tversky, 1992; Shekhar & Rahnev, 2021; Zhang & Maloney, 2012). While the presence of these biases may paint a bleak picture, the important fact is that subjective probability judgments are correlated with the likelihood of being correct (Baranski & Petrusic, 1998; Pleskac & Busemeyer, 2010; Vickers, 2014), which is referred to as the resolution of confidence (Murphy, 1972). Therefore, although subjective probability judgments are contaminated by several cognitive biases, the uncertainty they convey provides valuable information that can be leveraged to improve the quality of crowdsourced labels.

Biases in subjective probability judgments can be addressed by using cognitive models, such as the linear in log odds (LLO) function (Birnbaum & McIntosh, 1996; Turner et al., 2014; Tversky & Fox, 1995), to correct these biases. This function recalibrates subjective probabilities by transforming them into probabilities more aligned with objective truth. The two free parameters in this function have cognitive interpretations related to a response bias and over/underconfidence in subjective probability judgments (Gonzalez & Wu, 1999). Therefore, the LLO function recalibrates subjective probability judgments by minimizing biases present in these judgments.

We evaluate the efficacy of cognitive-inspired data engineering using the task of classifying peripheral blood cells, a critical step in the diagnosis of malignant blood diseases such as leukemia and lymphoma. While this is a medical decision-making task, our research objective is not limited to the medical domain. We are broadly interested in high-skill perceptual decision-making tasks and in developing AI systems for them. The task of classifying blood cells is an excellent sandbox for studying these questions because (1) it uses naturalistic images from an important real-world task, (2) it is simple enough that novices can achieve approximately 65% accuracy with minimal training (Trueblood et al., 2018), (3) it is difficult enough to challenge experts, and (4) it is possible to train ML models on the task with only a few hundred images (rather than thousands that are required for similar tasks). For these reasons, we believe this task and the associated images best suit the current work.

**Fig. 1:**
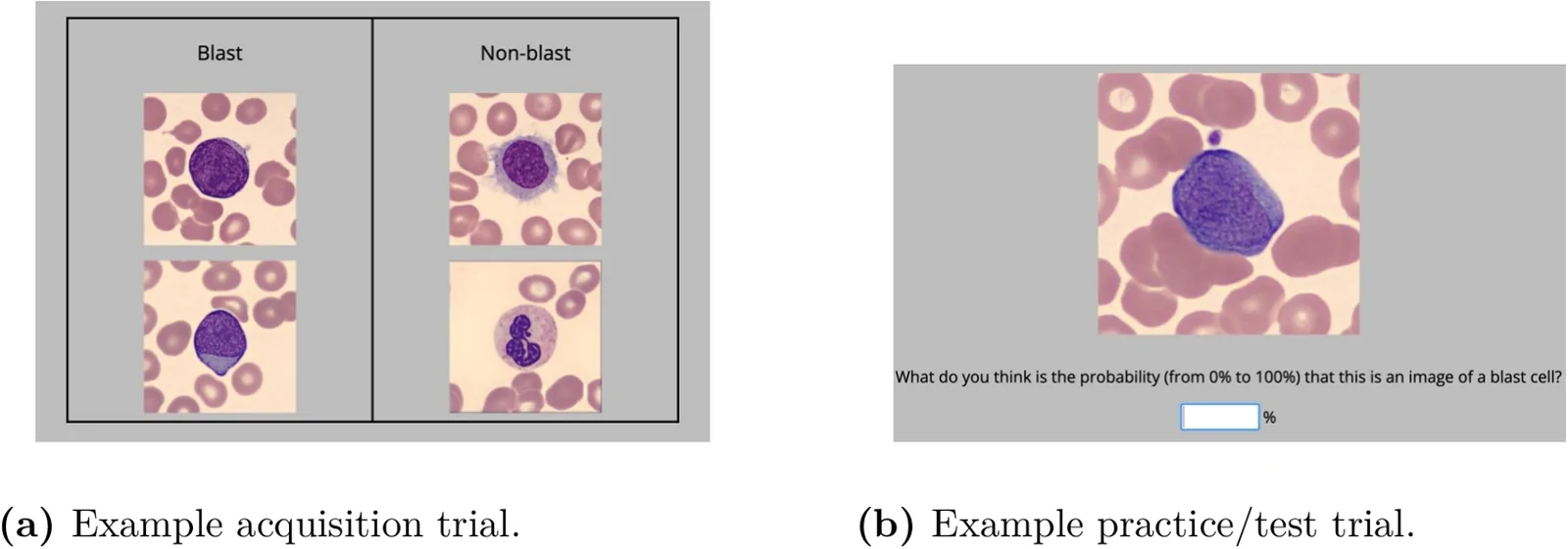
Example trials in Experiment 1. Each image contains a white blood cell, centered in the image, surrounded by red blood cells. Panel (**a**) shows an example acquisition trial where participants view four images for as long as they like. Panel (**b**) shows an example of a practice/test trial where participants provide probability judgments on a single image

While the experiments reported here use a medical image classification task, the paper’s central focus is the annotation-level recalibration mechanism rather than the task details. Prior work demonstrates that systematic biases in human probabilistic judgments arise across domains and can be corrected via recalibration. For example, Turner et al. (2014) show that recalibration improves aggregated probability forecasts in large-scale geopolitical forecasting tasks, and Han and Budescu (2022) demonstrate similar benefits across multiple economic forecasting domains, including inflation, GDP growth, and unemployment. These findings suggest that the recalibration principles employed here are not specific to medical imaging, but operate on general properties of subjective probability judgments.

In this article, we focus on recalibrated probability judgments as an example of cognitive-inspired data engineering. We evaluate whether recalibrated probability judgments, compared to uncalibrated probability judgments and binary choices, lead to a more accurate ML training dataset and whether a more accurate training dataset leads to a more accurate ML model. We investigate the efficiency of the labels by comparing the accuracy of the training sets and models as a function of the number of annotations per image. Finally, we examine how the number of judgments used to train the LLO function impacts the accuracy of the crowd-labeled datasets.

Our analyses are carried out over two experiments. Experiment 1 crowdsources annotations from minimally trained novices recruited through MTurk. Experiment 2 tests to see if the findings from Experiment 1 generalize to a real-world setting, where skilled annotators are recruited through DiagnosUs (Press, 2021), a crowdsourcing platform designed specifically for medical data annotation.

## Experiment 1

### Behavioral methods

#### Participants

A total of 400 Amazon Mechanical Turk (MTurk) workers participated in Experiment 1. Participants were recruited from MTurk through CloudResearch (Hauser et al., 2023; Litman et al., 2017), a crowdsourcing data acquisition platform. Across all participants, 61.25% of participants identified as a woman (N=245), 37% of participants identified as a man (N=148), 1% of participants identified as other (N=4), and .5% of participants preferred not to list their gender identity (N=2). We did not receive the demographic information from one of the participants. The mean age was 44.0 (SD=13.1; IQR = 34.0–53.0).

All participants who completed the 25-min experiment were paid a $1 base rate and had the potential to earn a $5 bonus to incentivize effort. The probability that a participant would receive the bonus was computed using the binarized, quadratic scoring rule (Brier, 1950; Selten, 1998). Overall, 72.25% of participants (N=289) received the $5 bonus.

First, any trial with a response time of 180 s or greater was excluded from the data analysis. Overall, .0125% of test trials were excluded (N=6). Next, participants were excluded from the data analysis if their mean response time on testing trials was either in the fastest 5% or the slowest 5% across participants. In total, 10% of participants (N=40) were excluded. After the exclusions, 360 participants remained.

#### Materials

The stimuli were 549 digital images of Wright-stained white blood cells taken from anonymized patient peripheral blood smears at Vanderbilt University Medical Center (VUMC) used in Trueblood et al. (2018). The images were taken by a CellaVision DM96 automated digital cell morphology instrument (CellaVision AB, Lund, Sweden). Three hematopathology faculty members from the Department of Pathology, Microbiology and Immunology at VUMC independently classified each image and rated the classification difficulty of each image on a scale of 1–5. We used only images with three-way classification agreement among curators and defined ground truth as the agreed-upon classification. Further details on how each image was classified and rated are in Trueblood et al. (2018).

Out of the 549 images, there were 266 blast images and 283 nonblast images. Figure 1 contains examples of both blast and nonblast images. Based on the mean difficulty rating provided by the three experts, images were partitioned into five difficulty categories (very easy, easy, medium, hard, and very hard). Out of all of the images, 30 blast images and 30 nonblast images were randomly selected to be part of a calibration set. We randomly sampled images so the expected number of images in the calibration set was equal across difficulty categories and, within each category, each image was equally likely to be sampled. This random sampling approach was used throughout Experiment 1 and will be referred to as equal-difficulty random sampling going forward. Collectively, there are 60 images in the calibration set (30 blast and 30 nonblast) and 489 images in the non-calibration set (236 blast and 253 nonblast images).

Out of the 30 blast images in the calibration set, four were in the very easy category, eight were in the easy category, eight were in the medium category, four were in the hard category, and six were in the very hard category. Out of the 30 nonblast images in the calibration set, three were in the very easy category, five were in the easy category, seven were in the medium category, eight were in the hard category, and seven were in the very hard category. Out of the 236 blast images in the non-calibration set, 35 were in the very easy category, 61 were in the easy category, 60 were in the medium category, 55 were in the hard category, and 25 were in the very hard category. Out of the 253 nonblast images in the non-calibration set, 58 were in the very easy category, 62 were in the easy category, 42 were in the medium category, 33 were in the hard category, and 58 were in the very hard category.

#### Procedure

There were two conditions in Experiment 1, manipulating the type of feedback participants received during practice: an accuracy feedback condition and an accuracy plus calibration feedback condition. In both conditions, participants first read instructions explaining that they would be presented with images of white blood cells and that their task would be to judge the likelihood that each image contained a blast cell.

Following the initial instructions, participants in both conditions completed an acquisition phase which consisted of 16 trials with four labeled images per trial. In each trial, participants were simultaneously shown two blast images on the left side of the screen and two nonblast images on the right side of the screen. For an example acquisition trial, see Fig. 1a. The images were randomly selected using equal-difficulty random sampling from the non-calibration set with the constraint that the same image was never presented twice on a single trial. These trials allowed participants to identify both commonalities within each image category as well as differences across the image categories. Participants were allowed to examine the images for as long as they wanted before proceeding to the next trial.

Next, participants in both conditions completed a practice phase which consisted of 24 trials. In each trial, participants were shown a single image and asked “What do you think is the probability (from 0% to 100%) that this is an image of a blast cell?”. Feedback was provided after each trial. The images were randomly selected using equal-difficulty random sampling from the non-calibration set with the constraint that half the trials were blast images, the other half were nonblast images, and no image was repeated during the practice phase.

In the accuracy feedback condition, participants received feedback only regarding whether the image was a blast cell. In the calibration feedback condition, participants received feedback on whether the image was a blast cell and on their likelihood of receiving the bonus, given their response and the image’s true class.

Finally, participants in both conditions completed a testing phase which consisted of five blocks with 24 trials per block. Similar to the practice phase, participants were asked “What do you think is the probability (from 0% to 100%) that this is an image of a blast cell?”. For an example practice/testing trial, please see Fig. 1b (note, the trial appearance is identical for practice and testing trials). Within a given block, ten of the images were blast images from the non-calibration set, ten of the images were nonblast images from the non-calibration set, two of the images were blast images from the calibration set, and two of the images were nonblast images from the non-calibration set. Each image was randomly selected using equal-difficulty random sampling, with the constraint that images were never repeated for a single participant across the entire testing phase. Feedback was not provided during the testing phase.

### Modeling methods

#### Recalibration method

Participants’ responses were calibrated using the linear in log odds (LLO) function, which is a technique used to transform subjective probabilities into probabilities that are more in line with objective truth (Birnbaum & McIntosh, 1996; Turner et al., 2014; Tversky & Fox, 1995). As shown in Eq. 1, the LLO function recalibrates judgments by first transforming a participant’s subjective probability judgments (denoted *p*) on images in the calibration set into log-odds values (denoted $p^{\prime }$):

1 $$\begin{aligned} p' = \ln {\bigg (\frac{p}{1-p}\bigg )}. \end{aligned}$$

Then, the log-odds values are used to fit a logistic regression model (Eq. 2) using the true labels for the images in the calibration set:

2 $$\begin{aligned} f(p) = \frac{1}{1 + e^{-(\alpha p' + \beta )}}. \end{aligned}$$

The LLO function can also be written as in Eq. 3:

3 $$\begin{aligned} \ln {\bigg (\frac{f(p)}{1-f(p)}\bigg )} = \alpha \ln {\bigg (\frac{p}{1-p}\bigg )} + \beta . \end{aligned}$$

**Fig. 2:**
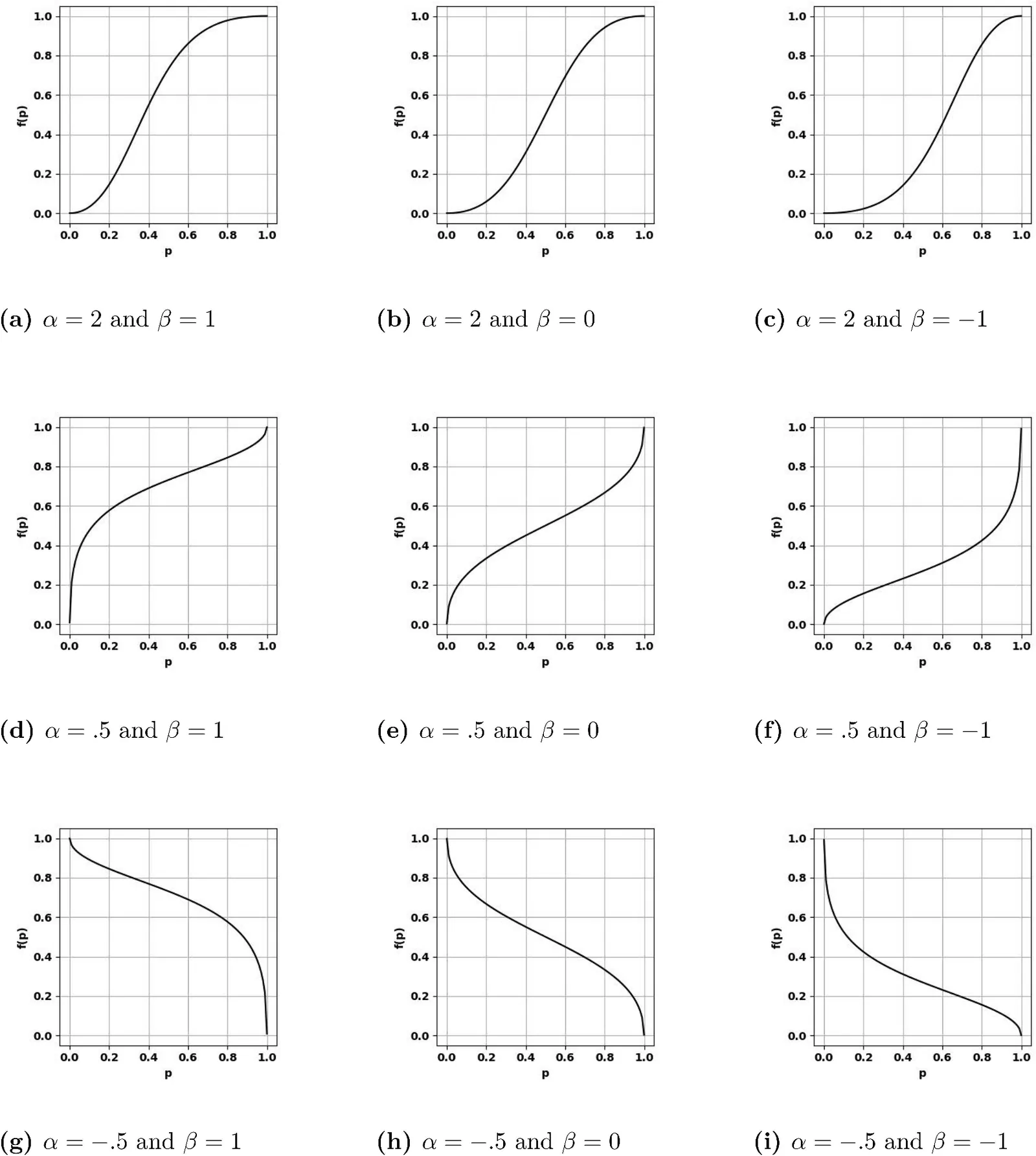
Form of the LLO function for combinations of parameters when $\alpha \in \{2,.5, -.5\}$ and $\beta \in \{1, 0, -1\}$. On the *x*-axis, *p* is the uncalibrated probability judgment and *f*(*p*), on the *y*-axis, is the recalibrated probability judgment

After fitting the logistic regression model, a participant’s responses on the non-calibration set are also transformed into log-odds values and the fitted logistic regression model is used to map these log-odds values into recalibrated probability judgments. Since a unique LLO function is fit to each participant’s responses in the calibration set, the recalibration transformation is unique to each participant. Recalibration is carried out separately for each annotator because the biases present in their judgments is subject to large individual differences (Baron et al., 2014; Pallier et al., 2002). The two parameters of the LLO function, α and β, each offer psychological interpretations regarding correcting biases in subjective probability judgments (Gonzalez & Wu, 1999). Figure 2 illustrates the form of the LLO function for several combinations of α and β.

The slope parameter (α) impacts the curvature of the LLO function, which corrects for biases due to over- or underconfidence in the raw subjective probability judgments. If |α|>1, the LLO function will increase the spread of the probability judgments, which indicates that a participant’s probability judgments were underconfident. For example, in Fig. 2b where α=2 and β=0, a probability judgment of p=20% is mapped to $f(p) \approx 5.9\%$ and p=80% is mapped to $f(p) \approx 94.1\%$, so the LLO function spreads the raw subjective probability judgments by pushing them towards the extreme ends of the probability scale. If |α|<1, the LLO function will decrease the spread of the probability judgments, which indicates a participant’s probability judgments were overconfident. For example, in Fig. 2e where α=.5 and β=0, a probability judgment of p=20% is mapped to $f(p) \approx 33.3\%$ and p=80% is mapped to $f(p) \approx 66.7\%$, so the LLO function constrains the raw subjective probability judgments by pulling them towards a more central point on the probability scale. To be precise, the LLO function pushes/pulls the judgments away from/towards $1/(1 + e^{\beta /\alpha })$. Note that, while the LLO function will always be monotonic, it will only be strictly increasing when α>0. We allow α to be less than 0 so that the LLO function can also be strictly decreasing. For example, in Fig. 2h where α=-.5 and β=0, a probability judgment of p=20% is mapped to $f(p) \approx 66.7\%$ and p=80% is mapped to $f(p) \approx 33.3\%$, so the LLO function flips the ordering of the probability judgments. This can be beneficial in cases where a participant is particularly poor at estimating the likelihood that an image contains a blast cell.

The intercept parameter (β) impacts the height of the LLO function, which corrects for biases in overall response tendency. If β>0, the LLO function will shift the distribution of probability judgments towards 1, which indicates that the participant was biased towards responding with low probabilities (underestimating the likelihood of blast and overestimating the likelihood of nonblast). For example, in Fig. 2d where α=.5 and β=1, a probability judgment of p=20% is mapped to $f(p) \approx 57.6\%$ and p=50% is mapped to $f(p) \approx 73.1\%$, so the LLO function increases the values of the subjective probability judgments. If β<0, the LLO function will shift the distribution of probability judgments towards 0, which indicates that the participant was biased towards responding with high probabilities (underestimating the likelihood of blast and overestimating the likelihood of nonblast). For example, in Fig. 2f where α=.5 and β=-1, a probability judgment of p=50% is mapped to $f(p) \approx 26.9\%$ and p=80% is mapped to $f(p) \approx 42.4\%$, so the LLO function decreases the values of the subjective probability judgments. Two special cases are when α=1 and β=0 and when α=0 and β=0. When α=1 and β=0, the LLO function reduces to the identity function. When α=0 and β=0, the LLO function maps all probability judgments to 50%.

Going forward, the raw subjective probability judgments will be referred to as elicited beliefs (EB) and the recalibrated subjective probability judgments will be referred to as recalibrated elicited beliefs (rEB).

#### ML training and hyperparameter selection

The 489 images in the non-calibration set are used for hyperparameter selection and model training/testing. For convolutional neural network (CNN) training and hyperparameter selection, we generated EB and rEB crowd labels for these images using five judgments per image, nine judgments per image, and all judgments per image. On average, each image received 73.6 judgments. When using all judgments per image, we first took the mean judgment for that image and then binarized the judgment at 50%: if the mean judgment was less than 50%, then the image was assigned a nonblast label, and if the mean judgment was greater than 50%, then the image was assigned a blast label. No images had a mean judgment of exactly 50% in the EB or rEB dataset. When using only a subset of judgments per image, we would randomly sample *N* (either 5 or 9) judgments, take the mean across those judgments, and then binarize the judgment at 50%. If the average judgment were 50% across the subset of judgments, we would randomly assign a blast or nonblast label (this occurred less than 1% of the time). We chose to create datasets using a subset of judgments on each image because anecdotal evidence from real-world medical data annotation tasks suggests that crowdsourced labels rarely exceed more than ten annotations per image, as crowd accuracy typically plateaus around ten annotations per image (Duhaime et al., 2023). Therefore, we have six different types of labeled datasets: EB with five judgments per image, EB with nine judgments per image, EB with all judgments per image, rEB with five judgments per image, rEB with nine judgments per image, and rEB with all judgments per image. For each type of labeled dataset, we generated 100 independent datasets by repeating the judgment-resampling procedure described above. Model training and evaluation were performed separately for each dataset.

For a given dataset, model training and evaluation proceeded as follows. The 489 images were partitioned into five folds, such that each fold contained approximately 20% of the images and preserved the relative proportions of blast and nonblast images. One fold was held out as a test set, and the remaining four folds were used for model training and hyperparameter selection. Candidate hyperparameter combinations were evaluated by training models on three folds and evaluating performance on the fourth fold, cycling through all four choices of validation subset. Validation performance was averaged across these splits, and the hyperparameters with the best average performance were selected.

Using the selected hyperparameters, a final model was trained on all four training folds and evaluated on the held-out test fold. This process was repeated five times such that each fold was the test fold exactly once, yielding exactly one held-out prediction per image for each dataset. Because this entire procedure was repeated independently for each of the 100 datasets, each image contributed 100 out-of-sample model predictions, one from each dataset, while maintaining strict separation between training, validation, and test data.

Each CNN was trained via transfer learning using PyTorch (Paszke et al., 2019). To implement this, we modified a GoogLeNet CNN (Szegedy et al., 2015) which was pre-trained on the ImageNet database using the same transfer learning procedure in Holmes et al. (2020). Each CNN was trained to minimize the cross-entropy loss between the CNN output and the training labels using the ADAM optimizer.

To identify the optimal hyperparameters for models trained on each type of labeled dataset, we performed a grid search over the number of training epochs (5, 10, and 15), learning rate ($1e^{-4}$, $5e^{-4}$, and $1e^{-3}$), L2-regularization strength ($1e^{-3}$ and $5e^{-3}$), and minibatch size (16 and 24). The results from the grid search over the candidate hyperparameters can be found in the supplementary material (supplementary Table 1).

**Fig. 3:**
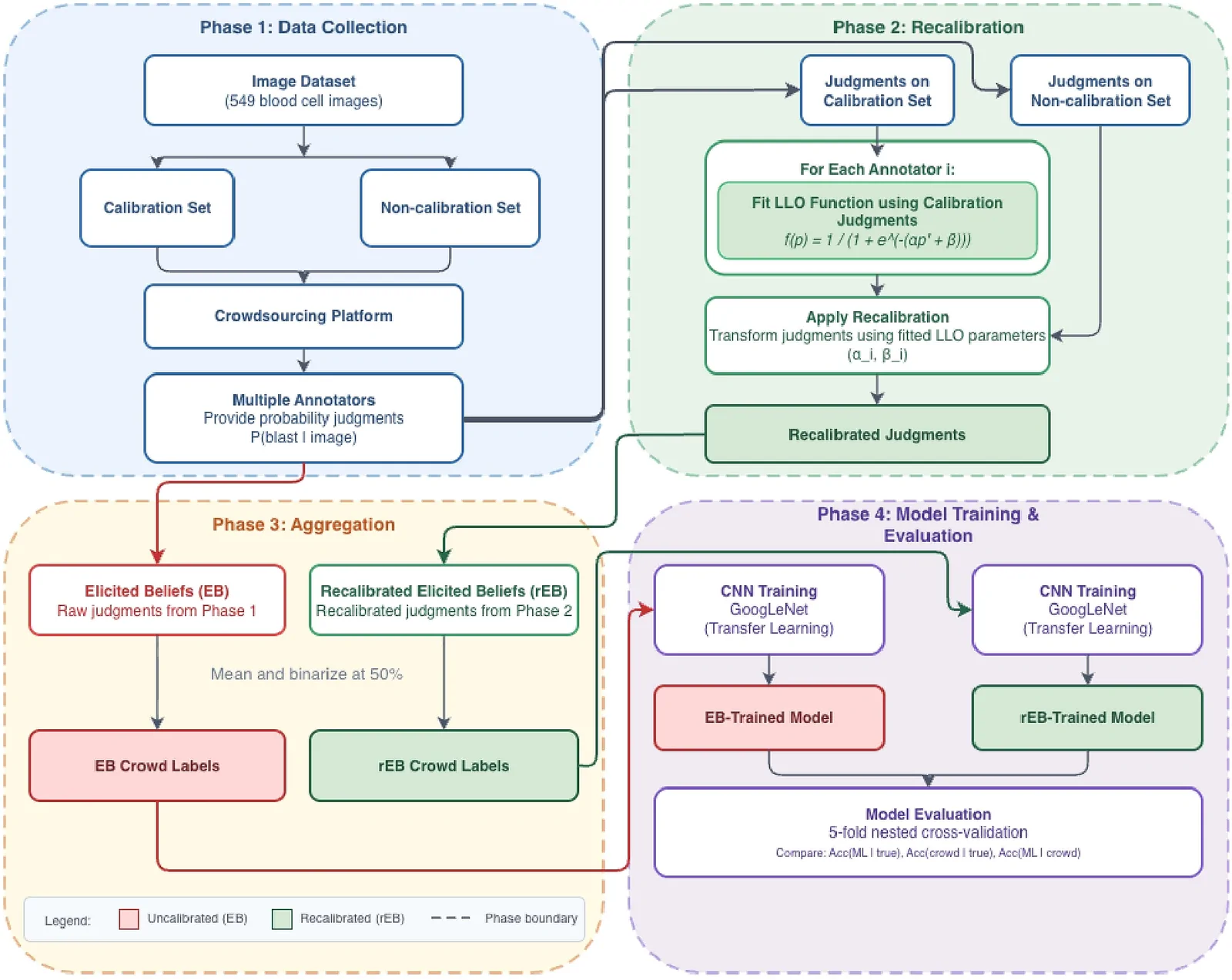
Study pipeline

Figure 3 illustrates the entire pipeline we employ to collect individual labels, recalibrate the labels, aggregate them into crowd labels, and train ML models on them. As illustrated in Fig. 3, the recalibration procedure operates entirely at the annotation level. Recalibration does not use any information from the machine learning models.

### Behavioral results

For Experiment 1, we combined the data across the two conditions because there were no apparent differences in participants’ performance. To verify this, we performed a two-sample Kolmogorov–Smirnov test comparing the distribution of participants’ accuracy on images in the non-calibration set in one condition versus the other and found no significant differences in the distributions of participants’ accuracy (p=.999). The distribution of participants’ accuracy in each condition can be found in the supplementary material (supplementary Fig. 1).

#### Individual performance

Accuracy was evaluated by binarizing participants’ probability judgments. Similar to how crowd labels were obtained by binarizing average judgments at 50%, accuracy was evaluated by first binarizing participants’ probability judgments and then comparing them to the true labels. As a result, a probability judgment was deemed correct (accuracy = 1) when either the image was a blast cell and the judgment was greater than 50% or the image was a nonblast cell and the judgment was less than 50%. Likewise, a probability judgment was deemed incorrect (accuracy = 0) when either the image was a blast cell and the judgment was less than 50% or the image was a nonblast cell and the judgment was greater than 50%. Since participants were allowed to respond with 50%, a 50% judgment was regarded as partially correct (accuracy = .5) regardless of whether the image was a blast cell or a nonblast cell, since there is no way to unambiguously binarize 50%.

**Fig. 4:**
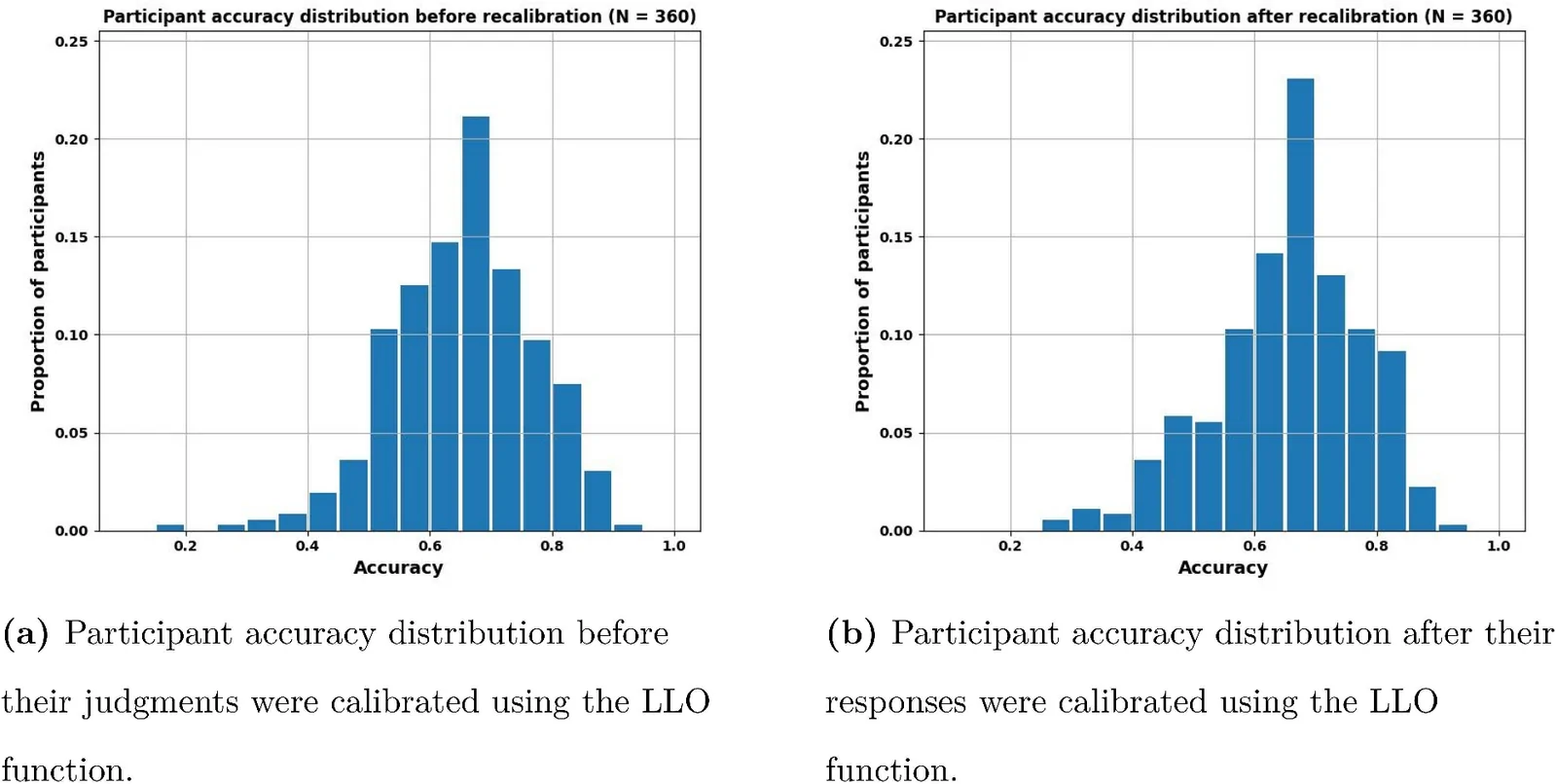
Participant accuracy distributions on images in the non-calibration set in Experiment 1

On average, participants were 65.3% accurate at classifying images in the non-calibration set before recalibrating using the LLO function and 65.4% accurate at classifying those images after recalibration. Figure 4 illustrates the distribution of accuracy scores across all participants before and after applying the LLO function to transform each participant’s responses.

**Fig. 5:**
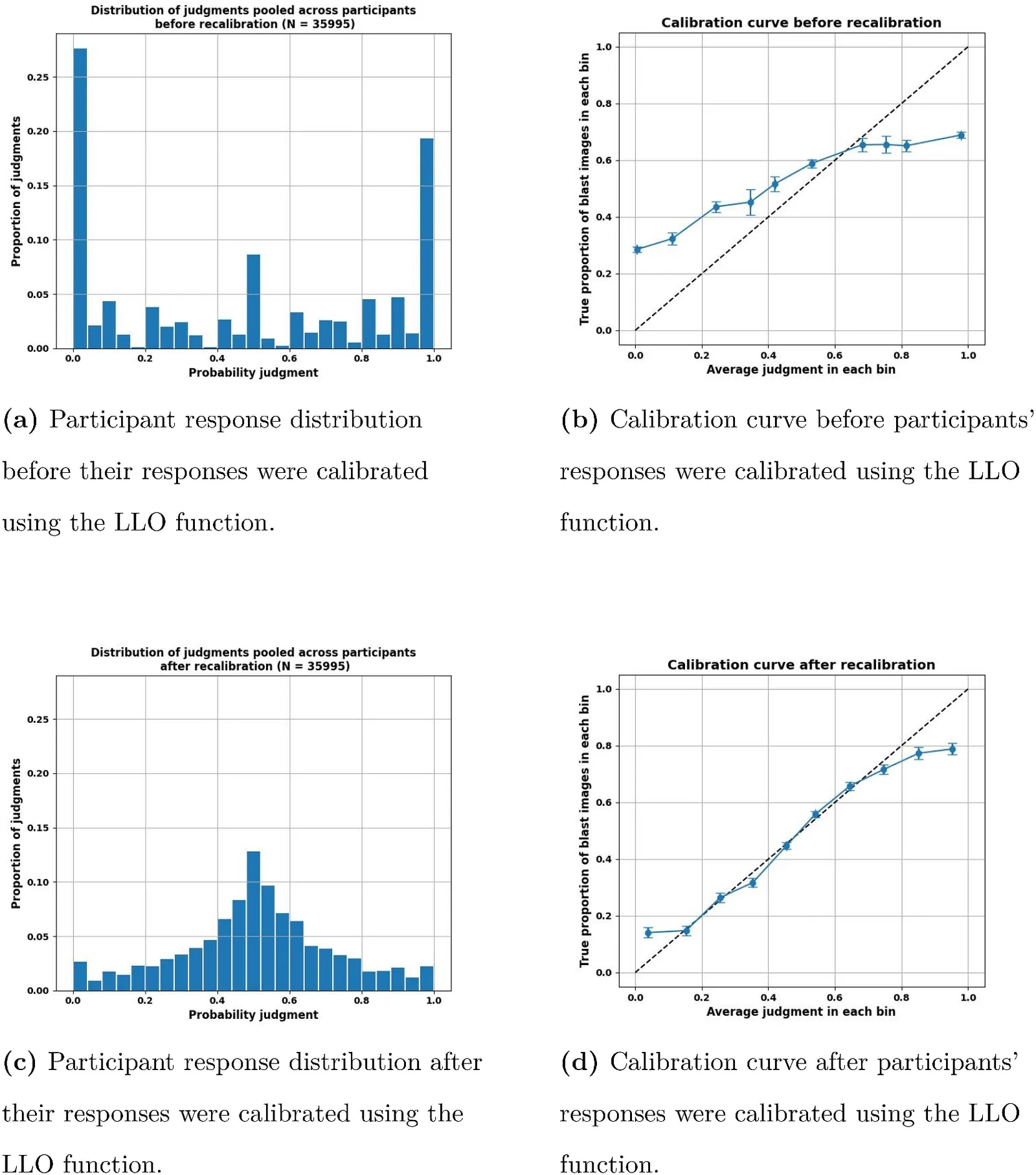
Participant response distribution and calibration curves on images in the non-calibration set in Experiment 1. For the calibration curves in the right panels, the *y*-error bars indicate the 95% confidence interval for the proportion of blast cells in each bin. There are also *x*-error bars indicating the 95% confidence interval for the mean judgment in each bin, but these intervals are so small that it is difficult to see the errorbars

While recalibration had a marginal impact on accuracy, it had a much stronger impact on the distribution of participants’ responses and the calibration of those responses. Figure 5 illustrates the distribution of subjective probability judgments pooled across all participants and the corresponding calibration curve before and after applying the LLO function to transform each user’s responses.

We generated the calibration curves by binning participants’ probability judgments into ten equally spaced bins. The *x*-value of the leftmost point on the calibration curve is the mean probability judgment in the first bin (mean judgment out of all judgments between [0, .1)) and the y-value is the proportion of images that were blast cells for the responses in that bin. Using the leftmost point in Fig. 5b as an example, the average probability judgment in that bin was 0% and, out of all the images that received a probability judgment between [0, .1), 28% of them were blast cells. The y=x line reflects a perfectly calibrated distribution because when a point of the calibration curve lies on that line, then the average probability judgment in a given bin is equal to the proportion of blast images in that bin. The further away the calibration curve deviates from the y=x line, the worse the calibration of the distribution is.

As shown in Fig. 5a, participants tended to favor the extreme ends of the probability scale, as well as responding 50%. Based on the calibration curve in Fig. 5b, it is evident that participants’ responses tended to be overconfident. Looking at the leftmost point of the calibration curve in that figure, when participants thought there was a 0% of an image being a blast cell, roughly 28% of the images were blast cells. Looking at the rightmost point of the calibration curve in the same figure, when participants thought there was a 100% chance an image contained a blast cell, only 68% those images contained a blast cell.

After applying the LLO function, participants’ judgments are transformed such that they are much more evenly distributed across the entire probability scale, with the majority of judgments being squeezed toward the center of the distribution (see Fig. 5c). Also, the calibration curve is now much closer to the y=x line. There is still some overconfidence at the extreme ends of the distribution in Fig. 5d, but much less so compared to Fig. 5b.

**Fig. 6:**
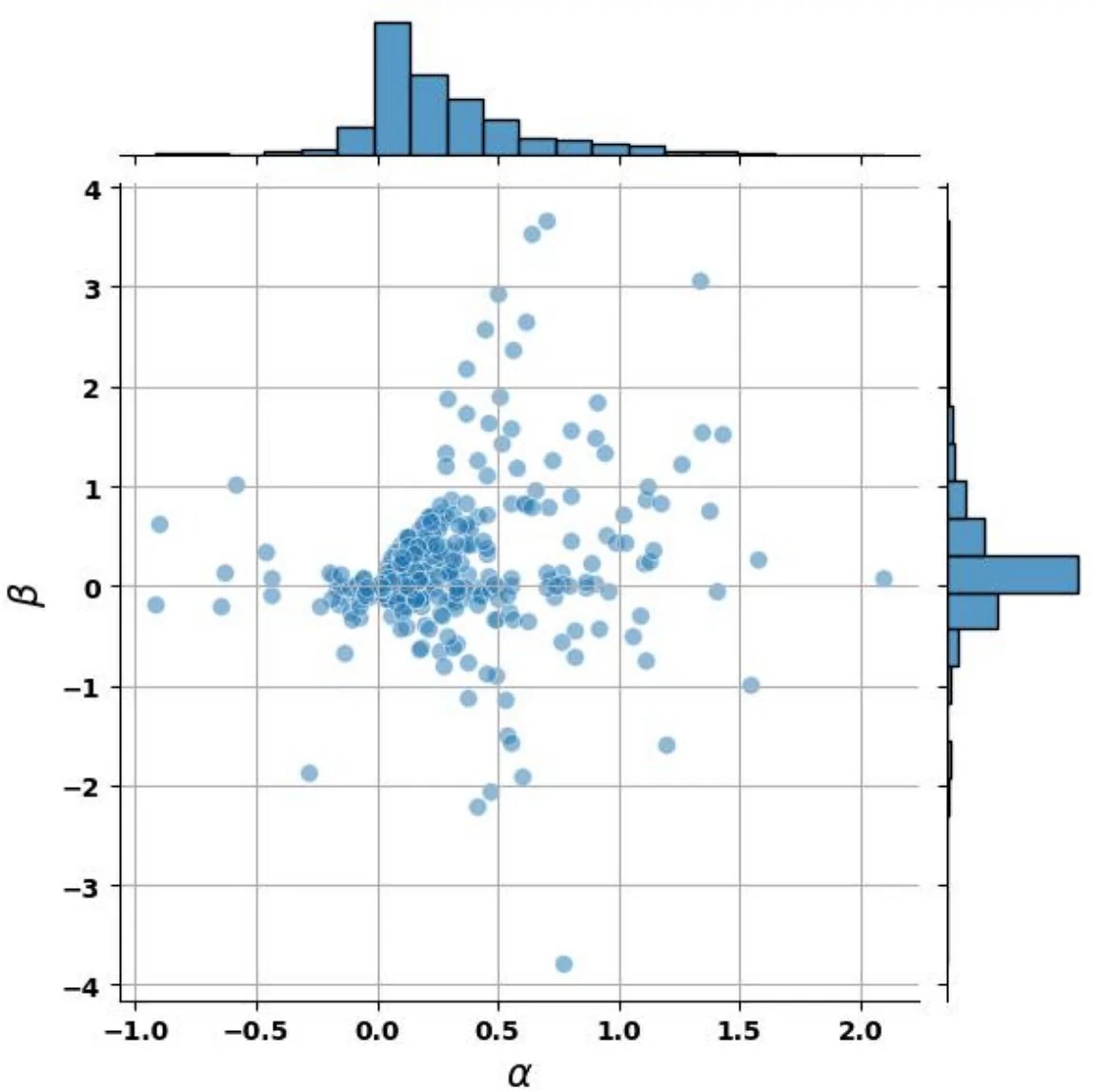
Best fit α and β parameters for the LLO function for participants in Experiment 1

Figure 6 depicts the distribution of the two parameters of the LLO function across all participants. The majority of the α parameters are between -1 and 1, indicating that most participants were overconfident in their judgments. This aligns with the changes in the calibration curve shown in Fig. 5, as the judgments were much more overconfident before recalibration as compared to after. Also, while most of the α parameters are greater than 0, a few are less than 0, indicating that the LLO function was strictly decreasing for some participants. This aligns with the individual accuracy plot (Fig. 4a), which shows that some participants had below-chance accuracy. Since some were exceptionally poor at discriminating between blast and nonblast images, the calibration of their judgments could be improved by flipping the ordering of their judgments, which is what the LLO function did using α<0.

**Table 1:** Crowd label accuracy on the non-calibration set in Experiment 1. For each dataset, one set of crowd labels is generated for each training and testing split. Since the ML models were evaluated using 100 unique sets of training and testing splits, the brackets indicate the 95% confidence intervals for the mean accuracy across the 100 sets of crowd labels. There are no brackets for the two datasets generated using all judgments per image because the label for each image does not vary across the sets of crowd labels

Dataset	Number of judgments per image	Accuracy	Accuracy on nonblast images	Accuracy on blast images
EB	5	.735 [.732, .739]	.777 [.773, .781]	.691 [.685, .697]
EB	9	.766 [.763, .769]	.800 [.795, .804]	.730 [.725, .735]
EB	all	.816	.830	.801
rEB	5	.778 [.775, .781]	.733 [.729, .738]	.826 [.821, .830]
rEB	9	.813 [.810, .816]	.756 [.752, .761]	.873 [.870, .876]
rEB	all	.851	.763	.945

The β parameters are centered at 0 and roughly symmetrically distributed, indicating that most participants did not display a strong response bias towards one end of the probability scale. However, there are a few outliers whose strong response bias was corrected by the LLO function.

In sum, the majority of participants learned to classify with above-chance accuracy, and therefore we can conclude that most successfully learned to distinguish between blast and nonblast cells. Although recalibration did not improve the accuracy of the participants’ judgments, it greatly improved their calibration, mainly by correcting overconfidence.

#### Crowd performance

Accuracy for the crowd labels was evaluated by binarizing average judgments at 50%, similar to how accuracy was evaluated for the participant’s probability judgments. First, we compared the accuracy of the EB crowd labels to that of the rEB crowd labels for the six different labeled datasets that will be used for ML training. Table 1 shows that recalibration improved the overall accuracy of the WoC labels from 81.6% to 85.1% when all judgments are used to generate the labeled dataset. When only five or nine judgments per image are used, the accuracy drops for both EB and rEB datasets, though the accuracy for rEB datasets always exceeds that of the EB datasets. Since recalibration led to improvements in crowd performance, but not individual performance, this suggests that the primary benefit of recalibration comes from altering the dispersion of probability judgments. By correcting overconfident annotators’ judgments by compressing them closer to 50%, recalibration reduces the impact of their judgments on the crowd label after binarizing. Similar, by correcting underconfident annotators’ judgments by pushing them to more extreme ends of the probability scale, recalibration increases the impact of their judgments on the crowd label after binarizing. Thus, recalibration functions as an implicit variance weighting mechanism at the group level.

**Fig. 7:**
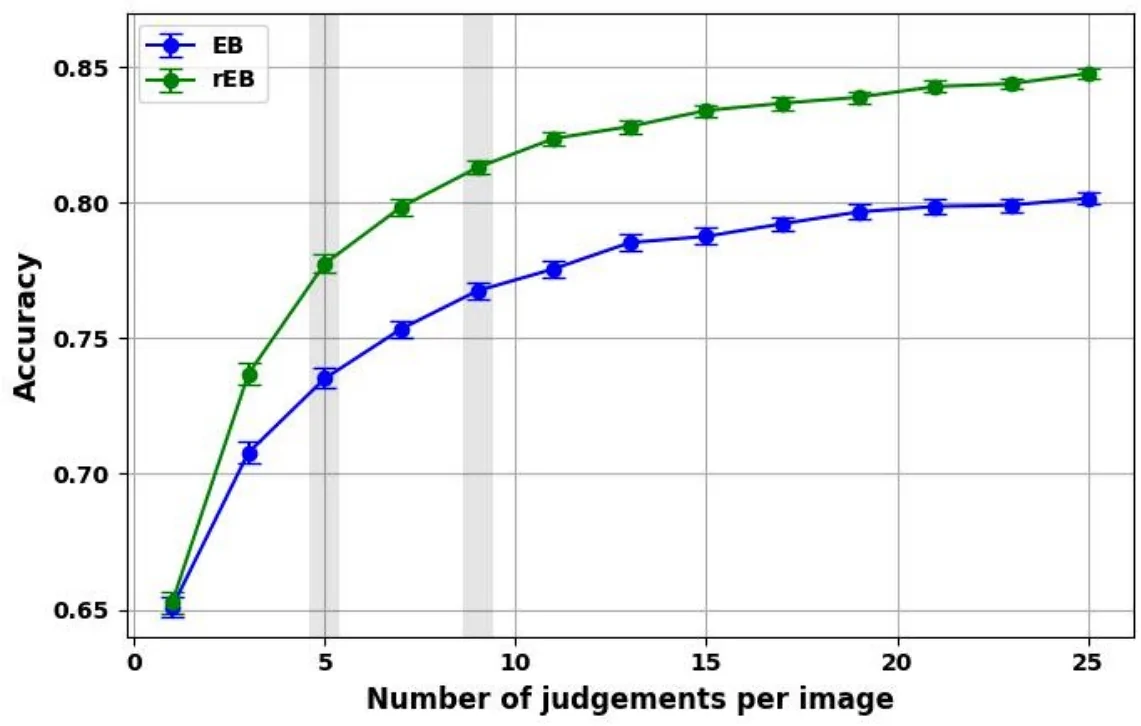
Crowd-label accuracy as a function of the number of judgments per image in Experiment 1. The *error bars* represent the 95% confidence interval for the mean accuracy across the 100 simulations for each value of *N*. The *two gray bars* highlight the crowd-label accuracy at five and nine judgments per image because these datasets are used in the ML model training and evaluation

Next, we further examined how the accuracy of the crowd labels changes as a function of the number of judgments per image. To do this, we randomly sampled *N* judgments from each image, generated the crowd label for each image, computed the accuracy, repeated this process for each image in the non-calibration set, and then took the mean accuracy across all images in the non-calibration set. *N* ranged from 1 to 25 judgments, and this process was simulated 100 times for each value of *N*. Figure 7 depicts the impact of the number of judgments per image on accuracy.

**Fig. 8:**
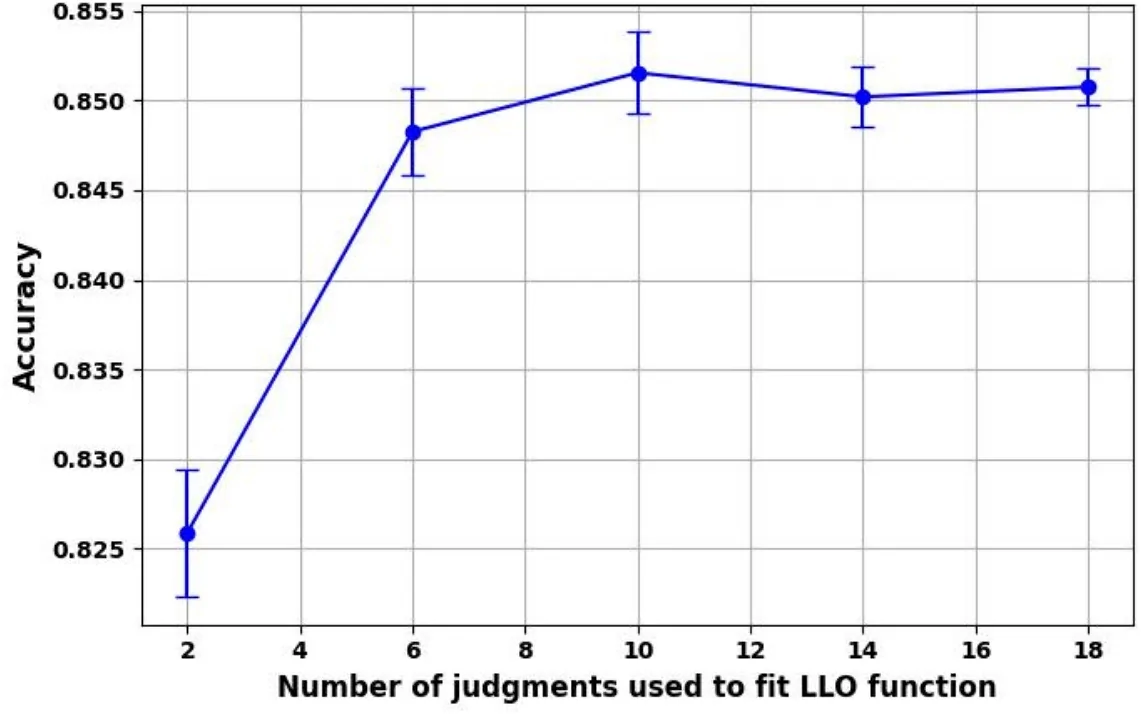
rEB crowd-label accuracy as a function of the number of judgments used to fit the LLO function in Experiment 1. The *error bars* represent the 95% confidence interval for the mean accuracy across the 100 simulations for each value of *N*

We see that not only do the rEB labels reach a higher level of accuracy compared to that of the EB labels, but the accuracy also initially increases at a quicker rate. Therefore, the rEB labels are more efficient in that they reach an arbitrary level of accuracy using fewer judgments.

Finally, we investigated how the number of judgments used to fit each participant’s LLO function impacted the rEB crowd accuracy in the non-calibration set. Recall, in the testing phase, participants made judgments on 20 images in the calibration set (ten blast and ten nonblast), and these judgments were used to calibrate the remainder of their judgments in the non-calibration set. To evaluate the impact of the size of the calibration set, we randomly sampled a set of *N* judgments on calibration images for each participant (where *N* is an even number such that there was always an equal number of judgments on blast and nonblast images), used these judgments to fit the LLO function and transform a user’s judgments on the non-calibration set, repeated this process for each user, and finally pooled the newly recalibrated judgments across users to obtain new crowd labels and evaluated the accuracy of these labels. *N* ranged from 2 to 18, and this process was simulated 100 times for each value of *N*. The results showing crowd label accuracy as a function of the number of judgments used for calibration can be found in Fig. 8.

**Table 2:** Mean accuracy on the training folds with 95% confidence intervals for Experiment 1

Dataset	Number of judgments per image	Acc(crowd | true)	Acc(ML| crowd)	Acc(ML | true)
EB	5	.735 [.733, .736]	.860 [.859, .862]	.800 [.797, .803]
EB	9	.765 [.763, .766]	.890 [.889, .892]	.815 [.813, .817]
EB	all	.816 [.815, .817]	.945 [.944, .946]	.839 [.838, .840]
rEB	5	.779 [.777, .780]	.887 [.885, .889]	.838 [.836, .841]
rEB	9	.814 [.812, .815]	.919 [.918, .921]	.852 [.850, .854]
rEB	all	.851 [.850, .851]	.967 [.966, .968]	.859 [.858, .861]

We were surprised to find that the benefit of the calibration set leveled off with as few as six judgments (three on blast images and three on nonblast images). To better understand why this is, we looked at the distribution of recalibrated judgments, calibration curves after recalibration, and distribution of α and β parameters using two, six, and ten judgments used to fit the LLO function. These figures can be found in the supplementary material (supplementary Figs. 2 and 3). With only two judgments, most of the LLO function parameters are clustered around α=0 and β=0, which is a special case of the LLO function that maps the raw judgments to 50%. Mapping the raw judgments to 50% effectively removes participants who performed poorly on the two judgments being sampled from the crowd label computation because the crowd labels are binarized at 50%. Since two judgments is a very small sample, the LLO function likely applies this mapping to judgments from well-calibrated and poorly calibrated individuals with a similar frequency. Unsurprisingly, the calibration curve using only two judgments looks very similar to that of the raw judgments since two judgments do not provide enough information for the LLO function to remove annotators’ responses biases. With six judgments, many responses are still mapped to 50%, but far fewer compared to with only two judgments. This is further supported by the distribution of LLO function parameters, with fewer parameter combinations clustering around α=0 and β=0. The resulting distribution of recalibrated judgments exhibits much more variability, and the calibration curve lies closer to the y=x line. With ten judgments, the variability of the distribution of recalibrated judgments further increases, and the calibration curve converges even closer to the y=x line, but the changes resulting from increasing the number of judgments from 2 to 6 is greater than the changes resulting from increasing the number of judgments from 6 to 10. This aligns with Fig. 8 where the crowd label accuracy increases by 2.2 percentage points (from 82.6% to 84.8%) when the number of judgments used to fit the LLO function increases from 2 to 6, but increases by only 0.4 percentage points (84.8% to 85.2%) when the number of judgments increases from 6 to 10. Therefore, with a sample size of six judgments, there’s enough information for the LLO function to effectively down-weight the judgments of poorly calibrated individuals in the crowd label computation, without doing the same to judgments from well-calibrated individuals. This demonstrates that even a very small calibration set can potentially yield marked improvement in crowd label accuracy.

### The effect of data re-calibration on CNN performance

We wanted to know whether differences in accuracy on the crowd-labeled datasets translated to the ML models trained on them. Note, throughout these analyses, the machine learning models serve as a diagnostic tool for assessing improvements in data quality, rather than as a component of the optimization procedure itself. The results of the model training can be found in Table 2 and the results for model testing can be found in Table 3. In both tables, Acc(crowd | true) is the accuracy of the crowd labels with respect to the true labels, Acc(ML | crowd) is the accuracy of the ML model with respect to the crowd labels, and Acc(ML | true) is the accuracy of the ML model with respect to the true labels. Since accuracy is usually defined with respect to ground truth, it may be helpful to think of Acc(X | Y) as the proportion of images where X and Y agreed on the same label.

**Table 3:** Mean accuracy on the testing folds with 95% confidence intervals for Experiment 1

Dataset	Number of judgments per image	Acc(crowd | true)	Acc(ML| crowd)	Acc(ML | true)
EB	5	.735 [.731, .738]	.731 [.727, .735]	.811 [.807, .815]
EB	9	.765 [.761, .768]	.773 [.769, .777]	.827 [.823, .830]
EB	all	.816 [.813, .819]	.858 [.854, .862]	.860 [.857, .863]
rEB	5	.779 [.775, .782]	.777 [.773, .780]	.849 [.845, .853]
rEB	9	.814 [.811, .817]	.822 [.819, .826]	.860 [.857, .864]
rEB	all	.851 [.849, .852]	.893 [.891, .896]	.869 [.865, .873]

First, Acc(crowd | true) increases as the number of judgments per image increases in both tables. Note, Acc(crowd | true) only differs across the two tables due to random sampling because the images were randomly divided into training and testing folds. Also, for a fixed number of judgments per image, the accuracy of the rEB labels is always greater than that of the EB labels. Both of these findings correspond to Fig. 7, which illustrates that the accuracy of the crowd labels increases as the number of judgments per image increases for both EB and rEB labels, and the rEB curve always lies above the EB curve.

Next, Acc(ML | crowd) increases as the number of judgments per image increases in both tables. Recall that the ML model is trained on crowd labels, which contain less noise as the number of judgments per image increases. As the amount of noise in the labels decreases, the model can more easily align its output with the data it is trained on. Therefore, Acc(ML | crowd) increases as the number of judgments per image increases because the amount of noise in the crowd labels decreases.

We also notice that, for a fixed number of judgments per image, Acc(ML | crowd) is always higher for the rEB labels compared to the EB labels. The LLO function counteracts biases in subjective probability judgments, and both the degree and the direction of these biases are subject to individual differences. By reducing these biases in subjective probability judgments, the LLO function serves as a way to normalize the judgments across annotators before combining them into crowd labels. From this perspective, LLO calibration can be viewed as another form of data pre-processing (similar to min-max scale, *z*-score normalization, etc.) but specific to this type of data (García et al., 2016). Therefore, the set of rEB labels contains less noise due to annotators’ biases compared to EB labels because participants’ judgments are semi-normalized by the LLO function. As a result, compared to the EB labels, the rEB labels are easier for the ML model to learn at a fixed number of judgments per image.

Unlike Acc(crowd | true), Acc(ML | crowd) can differ across the two tables. More specifically, if Acc(ML | crowd) is greater for the training set than the testing set, this is a sign that the models are overfitting to the training labels. We see that Acc(ML | crowd) is much higher in the training folds compared to the testing folds at a fixed number of judgments for both EB and rEB datasets. This is expected given that we are working with a very small dataset, which makes it difficult to train a model without overfitting.

Lastly, Acc(ML | true) increases as the number of judgments per image increases in both tables and, at a fixed number of judgments, models trained on rEB labels outperform those trained on EB labels. These trends show that improvements in crowdsourced dataset quality from recalibration translate into improvements in ML model quality. Note, we are mainly interested in Acc(ML | true) in the testing folds since the overall objective is to maximize the accuracy of the ML model with respect to the true labels on out-of-sample images (which would be the accuracy of the model when deployed into production). Nevertheless, Acc(ML | true) differs very little across the training and testing folds.

## Experiment 2

For complex medical diagnostics tasks such as the one used in our experiments, companies will typically turn towards more specialized crowdsourcing platforms rather than using the more domain-general ones such as MTurk. Experiment 2 extends Experiment 1 into a ‘real-world’ setting by using the same task of classifying white blood cells but performed through DiagnosUs, a free publicly available app on the IOS App Store produced by Centaur Diagnostics Inc. Press (2021), which is a crowdsourcing platform that specializes in medical data annotation. Using this platform, we evaluate our cognitive-inspired data engineering approach of recalibrating individual probability judgments with the aim of improving crowdsourced data accuracy.

The key to our recalibration approach is the inclusion of a holdout set of images used to fit a recalibration model that can then be used to correct cognitive biases on the remaining judgments. DiagnosUs presents labeling tasks to annotators in the form of contests and users can choose whether to participate in each contest. In a contest, gold standard (GS) images (images with ground truth labels) are randomly shuffled in with unlabeled images to provide feedback to users and continually assess their performance. Since these are images for which there are existing ground truth labels, GS images serve as a natural calibration set. Thus, we are able to leverage the existing task structure on DiagnosUs for our recalibration approach.

### Behavioral methods

#### Participants

A total of 175 participants were recruited through DiagnosUs for Experiment 2. We have some demographic information regarding geographic location and occupation for 85 out of the 175 participants. Regarding the geographic location of the 85 participants for whom we have some demographic information, 23 were from Asia, 22 were from Africa, 12 were from North America, six were from Europe, 1 was from South America, and 21 did not provide their geographical location. Regarding the occupation of those same participants, 32 were medical students, 21 were not in a profession related to the medical field, 12 were other healthcare students, three were pharmacy students, three were medical doctors, two were nurse practitioner students, one was a physician assistant student, one was a nurse practitioner, and ten did not provide their occupation.

Participants competed to win prize money, which was determined by their position on the contest leaderboard. Their place on the leaderboard was determined by their performance in the contest. In order to be eligible for the prize, participants had to complete at least 200 competition trials. Responses from users who completed fewer than 200 trials were excluded from further analysis. In each condition, first place received a $30 prize, second place received a $25 prize, third place received a $20 prize, fourth and fifth place received a $15 prize, sixth place received a $10 prize, seventh place received a $5 prize, and eighth through twelfth place received a $1 price. All other participants received no prize money.

#### Materials

The same 549 images of Wright-stained white blood cells used in Experiment 1 were also used in Experiment 2. The images were randomly divided into a quality assurance (QA) set containing 300 images (150 blast and 150 nonblast images) and a gold standard (GS) set containing 249 images (116 blast and 133 nonblast images). Splitting the data into GS and QA sets is standard practice due to the incentive structure of the DiagnosUs app. The distribution of the mean difficulty ratings for images in the GS and QA sets is included in the supplementary materials (supplementary Table 2).

#### Procedure

There were two conditions in Experiment 2, which differ based on the type of response elicited from participants: an elicited beliefs (EB) condition and a binary choice (BC) condition. For binary classification tasks, the industry-standard method of eliciting responses from annotators is to elicit binary choices (indicating one class or the other), rather than probability judgments (Collins et al., 2022; Duhaime et al., 2023). We included the binary choice condition to better compare our approach to the industry-standard elicitation method in terms of the accuracy of both the crowdsourced datasets and the models trained on these datasets. To ensure participants did not compete in both conditions, we randomly divided the app’s user base such that half of the users were only presented with the EB version of the contest and the other half of the users were only presented with the BC version of the contest. A total of 97 users participated in the BC condition, and 78 users participated in the EB condition.

**Fig. 9:**
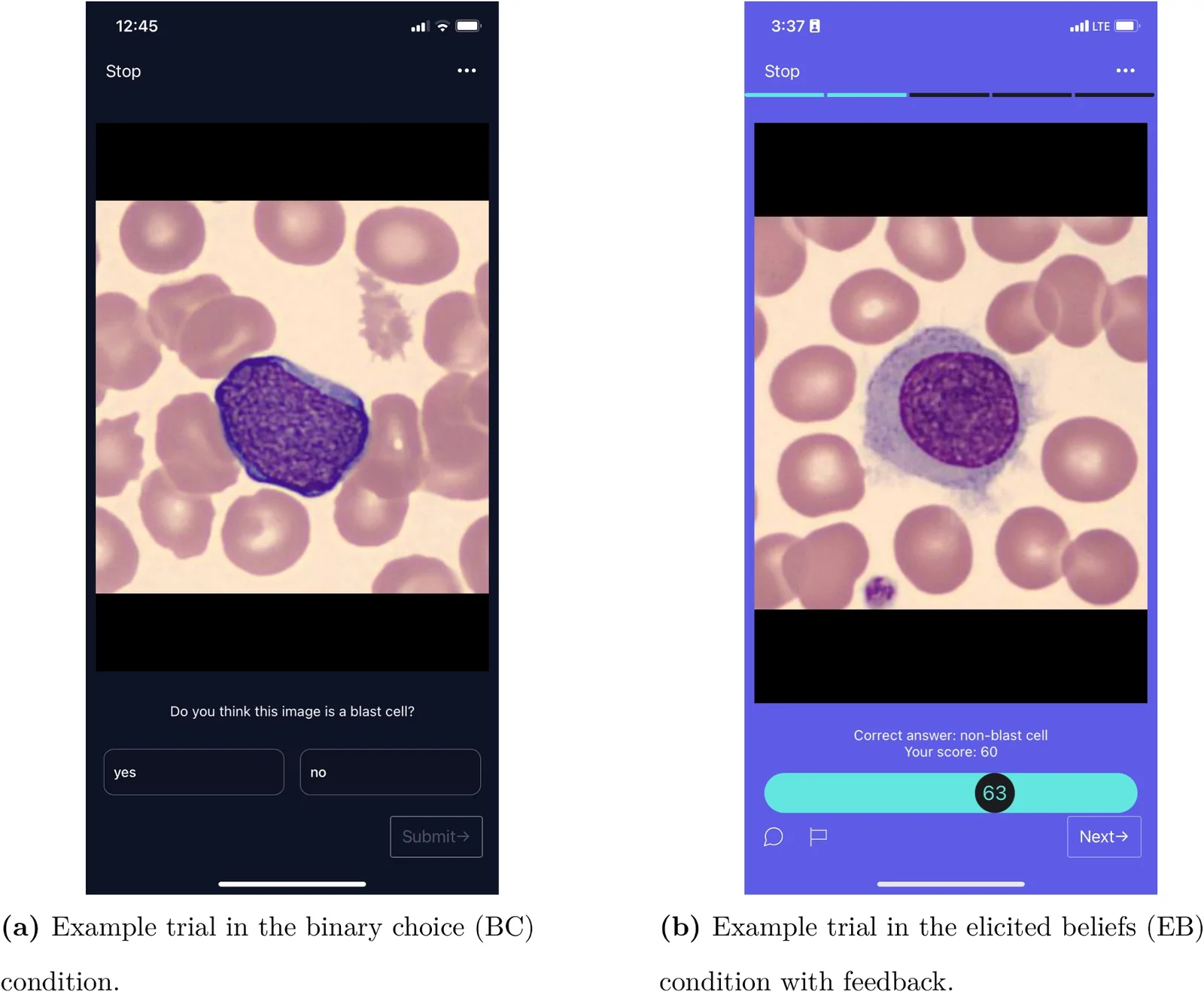
Example trials in Experiment 2 on the DiagnosUs app platform

In both conditions, participants first read instructions explaining that they would be presented with images of white blood cells. In the EB condition, participants were informed that their task would be to judge the likelihood that each image contained a blast cell. In the BC condition, participants were informed that their task would be to determine whether an image contained a blast cell. After the initial instructions, participants completed 8 practice trials to familiarize themselves with the task. On each practice trial in the EB condition, participants were asked, “What is the likelihood that this is a blast cell?”. Participants entered their response using a slider mechanism and were given feedback in terms of the correct answer and a score, which was computed using the binarized, quadratic scoring rule. Similarly, on each practice trial in the BC condition, participants were asked “Do you think this image is a blast cell?” (Fig. 9). Participants would either select ‘yes’ or ‘no’ and they were provided feedback (just the correct answer) after each response. Images in the practice trials were randomly sampled from the GS set with replacement.

After the eight practice trials, participants were free to complete as many competition trials as they would like. The answer prompts and response-elicitation mechanisms in the competition trials were identical to those used in the practice trials. For both practice and competition trials, there was no time limit set for each trial so participants could take as long as they liked to make their judgment. However, any response on a competition trial that took longer than 3 min was excluded from further analysis, similar to Experiment 1. Images in the competition trials were randomly sampled such that 1/3 of the images came from the GS set and 2/3 of the images came from the QA set, with the constraint that no user saw the same image twice within a 24-h period and that it was equally likely for a blast or nonblast cell to be sampled on each trial. Participants received feedback on competition trials in the same format as the practice trials, but only on trials where images were sampled from the GS set. No feedback was provided for responses on QA images.

In the EB condition, participants’ performance (and therefore their place on the leaderboard) was based on their average score on competition trials for images in the GS set. Similarly, in the BC condition, participants’ performance was based on their accuracy on competition trials for images in the GS set.

Because each contest ran for 48 h, it was possible for users to see the same image twice. If a user saw an image more than once, we only included their response in the data analysis from the first time they saw the image. This was done to ensure that each user contributed at most one judgment to each crowd label and to remove any confounds due to memory effects on a user’s decision-making process.

### Modeling methods

#### Recalibration method

In the EB condition, participants’ judgments for images in the GS set were used to recalibrate their judgments for images in the QA set using the LLO function, just as participants’ judgments for images in the calibration set were used to recalibrate their judgments for images in the non-calibration set in Experiment 1. Hence, we use the true labels to provide feedback to users and to fit the LLO function that transforms their judgments on images for which we aim to create crowd labels for ML model training.

#### ML training and hyperparameter selection

The 300 images in the QA set were used for hyperparameter selection and model training/testing. For the CNN training and hyperparameter selection, we generated BC, EB, and rEB crowd labels for these images using five judgments per image, nine judgments per image, and all judgments per image. On average, each image received 51.8 judgments in the EB condition and 62.4 judgments in the BC condition. EB and rEB crowd labels were created using the same process as described in Experiment 1. BC crowd labels were created by taking the majority judgment. If there were more ‘yes’ (blast) judgments than ‘no’ (nonblast) judgments for an image, then the image was assigned a blast label, and if there were more ‘no’ (nonblast) judgments than ‘yes’ (blast) judgments for an image, then the images was assigned a nonblast label. No image had an equal number of ‘yes’ and ‘no’ judgments. Therefore, we have nine different sources for our labeled datasets: BC with five votes per image, BC with nine votes per image, BC with all votes per image, EB with five votes per image, EB with nine votes per image, EB with all votes per image, rEB with five votes per image, rEB with nine votes per image, and rEB with all votes per image.

The hyperparameter selection and model training/testing procedure was nearly identical to Experiment 1, with the only difference being the set of images used (QA set here, whereas non-calibration set was used in Experiment 1). The results from the grid search over the candidate hyperparameters can be found in the supplementary materials (supplementary Table 3).

### Behavioral results

#### Individual performance

**Fig. 10:**
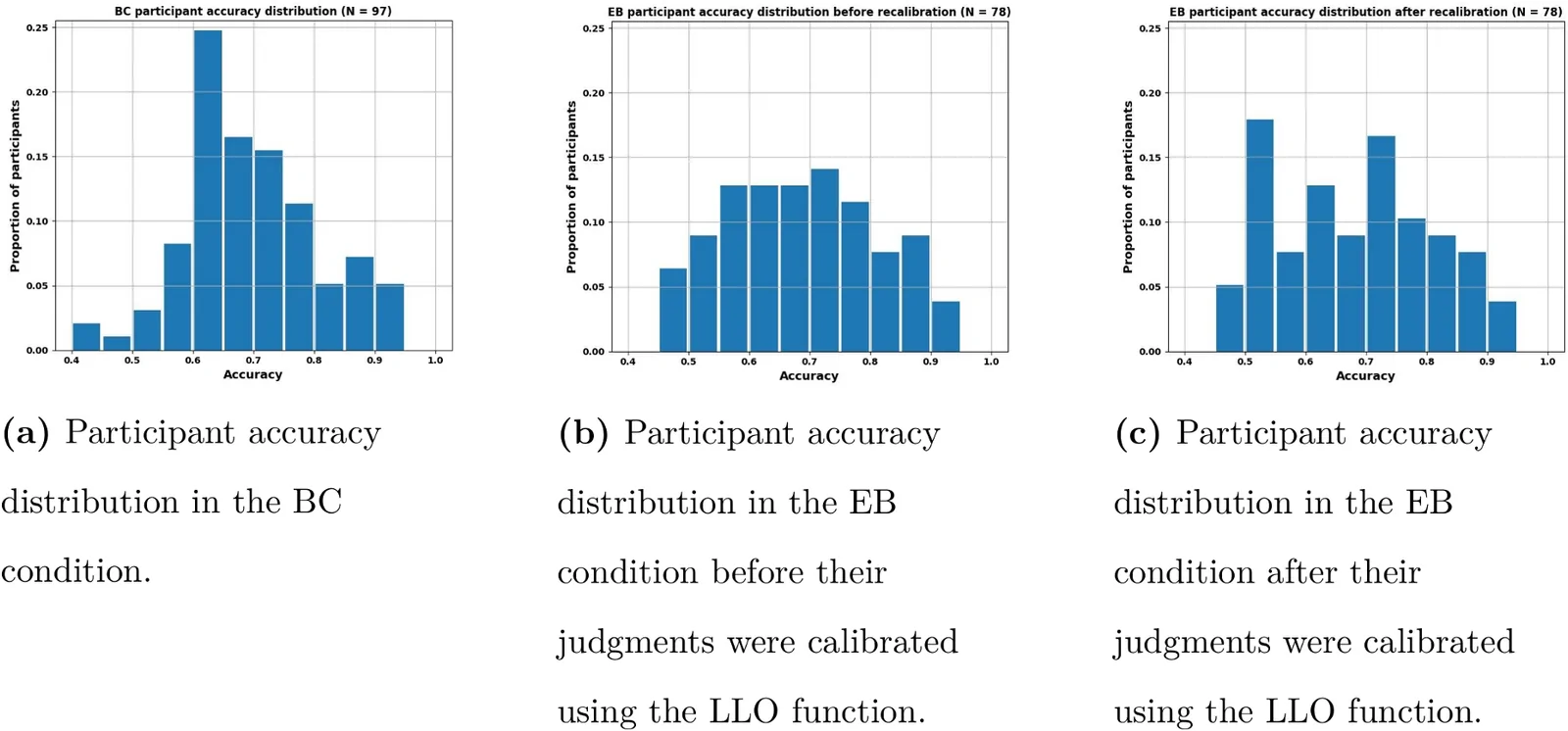
Participant accuracy distributions on images in the quality assurance (QA) set in Experiment 2

Similar to Experiment 1, accuracy was evaluated in the EB condition by binarizing participants’ probability judgments. On average, participants were 68.7% accurate in classifying images in the QA set before recalibrating using the LLO function and 68.2% accurate in classifying those images after recalibration. In the BC condition, participants were on average 69.8% accurate at classifying images in the QA set. Figure 10 illustrates the distribution of accuracy scores across all participants in the BC condition and before and after recalibration in the EB condition.

**Fig. 11:**
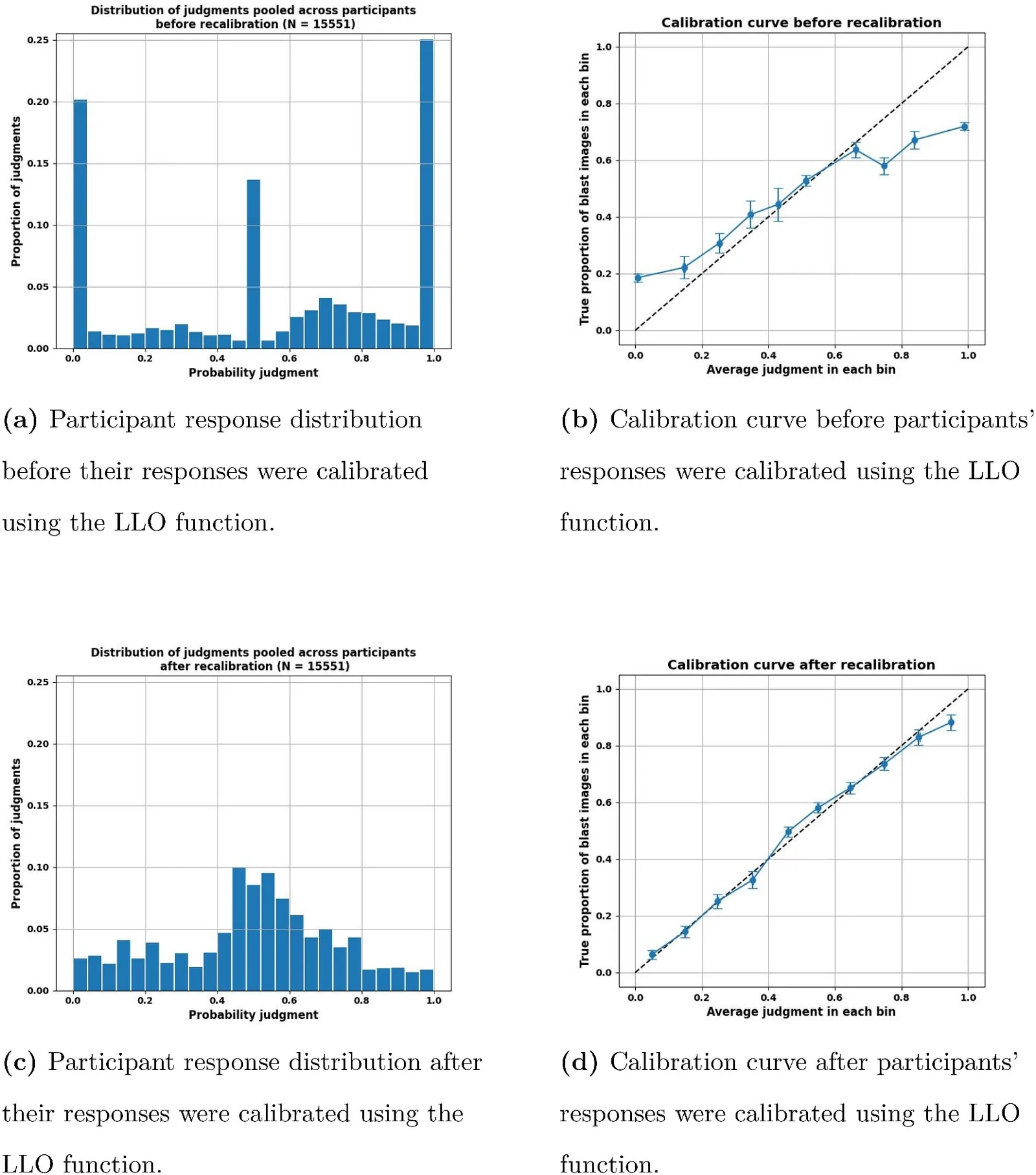
Participant response distribution and calibration curves on images in the QA set in the EB condition of Experiment 2. For the calibration curves in the right panels, the *y*-error bars indicate the 95% confidence interval for the proportion of blast cells in each bin. There are also *x*-error bars indicating the 95% confidence interval for the mean judgment in each bin, but these intervals are so small it is difficult to see the error bars

Similar to Experiment 1, recalibration had an extremely minor impact on accuracy, but had a much stronger impact on the distribution of judgments and the calibration of those judgments. Figure 11 illustrates the distribution of subjective probability judgments pooled across all participants and the corresponding calibration curve before and after applying the LLO function to transform each participant’s responses for images in the QA set.

Before recalibration, participants were heavily biased towards responding at the extreme ends of the probability scale and at exactly 50% as shown in Fig. 11a. As a result of this bias towards extreme responding, the calibration curve depicted in Fig. 11b illustrates that participants’ responses tended to be overconfident. Compared to the calibration curve before recalibration in Experiment 1 (see Fig. 5b), participants’ judgments were not as overconfident and were better calibrated in Experiment 2, especially judgments towards the middle of the probability scale, which were very well calibrated. Nonetheless, transforming the judgments using the LLO function led to a major improvement in calibration, as the calibration curve after recalibration in Fig. 11d is much closer to the y=x line compared to the curve before recalibration in Fig. 11b. Recalibration had a similar impact on the overall distribution of responses pooled across participants compared to Experiment 1, where the majority of responses were squeezed towards the middle of the probability scale.

**Fig. 12:**
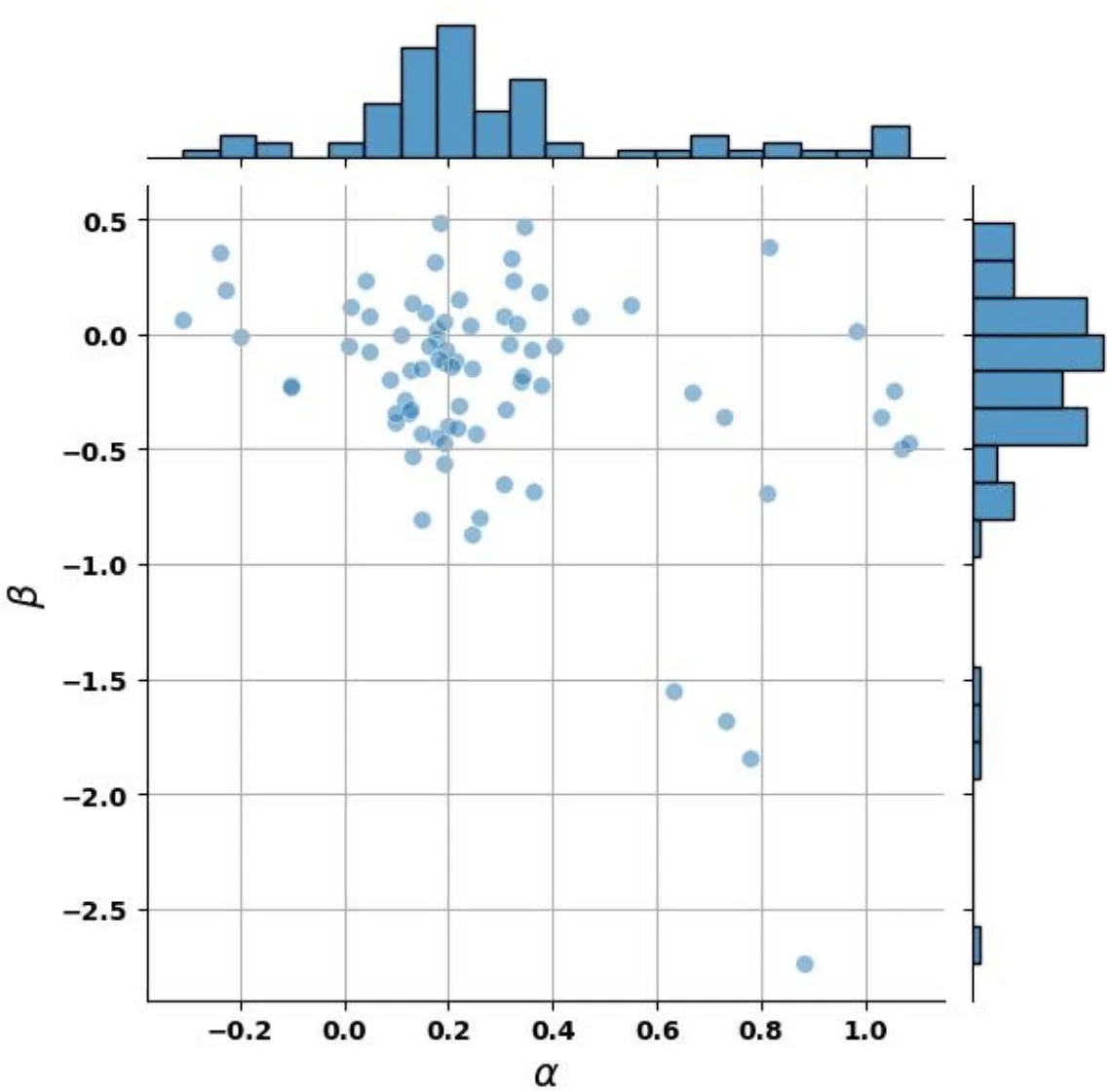
Best fit α and β parameters for the LLO function pooled across participants in Experiment 2

Figure 12 depicts the distribution of the two parameters from the LLO function across all participants in Experiment 2. Nearly all α parameters fall between –1 and 1, indicating that the majority of participants were overconfident in their judgments. This is a similar trend to that of Experiment 1, and also aligns with the changes in the calibration curve as a result of recalibration in Fig. 11 because the judgments were much more overconfident before recalibration compared to after. Only a few participants had α<0. This is not surprising given that most participants were above chance accuracy, meaning they did a fairly good job at discriminating between blast and nonblast images. Also, many more judgments were used to fit the LLO model in Experiment 2 (111.3 judgments, on average), so a participant was less likely to perform poorly on the calibration set of images due to random chance.

Unlike Experiment 1 where the β parameter was symmetrically distributed around 0, the β parameter is skewed in the negative direction, indicating that participants were biased towards responding with high probability (i.e., overestimated the likelihood of an image containing a blast cell). We believe this could result from the population of users on the DiagnosUs app. Out of the 75 users for whom we have demographic information and who provided information regarding their occupation, 65 of them ($\approx 86.7\%$) reported an occupation in the medical domain. In the medical domain, false positives are often viewed as less costly compared to false negatives. As a result, the users may have been more likely to err on the side of diagnosing a white blood cell as cancerous (a blast cell) when they were unsure of the true category of the image.

#### Crowd performance

Similar to Experiment 1, accuracy for the crowd labels was evaluated by binarizing the judgments at 50% for the EB and rEB datasets. Accuracy for the crowd labels in the BC datasets was evaluated by taking the more popular judgment for each image (i.e., majority rule) and comparing that label to the true label.

Table 4 contains the accuracy of the crowd labels for the datasets that are used for ML training. First, we compared crowd-label accuracy across conditions when all judgments were used to generate the labels. We found that the accuracy of the crowd labels in the EB and BC conditions was extremely similar, both 88.3% accurate. Recalibration had a very positive effect, increasing crowd accuracy from 88.3% to 96.7%. Similar to Experiment 1, with only five or nine judgments per image, the accuracy drops for BC, EB, and rEB datasets. Overall, crowd accuracy in Experiment 2 was higher compared to that of Experiment 1, but this was expected given that the MTurk participants in Experiment 1 are novices, whereas the participants on the DiagnosUs app are skilled annotators.

**Table 4:** Crowd-label accuracy on the non-calibration set in Experiment 2. Since the ML models were evaluated using 100 unique sets of training and testing splits, the *brackets* indicate 95% confidence intervals for the mean accuracy across the 100 sets of crowd labels. There are no brackets for the three datasets generated using all judgments per image because the label for each image does not vary across the sets of crowd labels

Dataset	Number of judgments per image	Accuracy	Accuracy on nonblast images	Accuracy on blast images
BC	5	.788 [.784, .792]	.680 [.673, .688]	.896 [.892, .900]
BC	9	.826 [.823, .829]	.706 [.700, .711]	.947 [.944, .950]
BC	all	.882	.780	.983
EB	5	.795 [.791, .799]	.674 [.667, .681]	.916 [.911, .920]
EB	9	.826 [.822, .829]	.692 [.687, .698]	.959 [.956, .963]
EB	all	.883	.767	1.000
rEB	5	.836 [.832, .840]	.811 [.805, .817]	.861 [.855, .867]
rEB	9	.890 [.887, .893]	.855 [.850, .860]	.925 [.920, .929]
rEB	all	.967	.940	.993

**Fig. 13:**
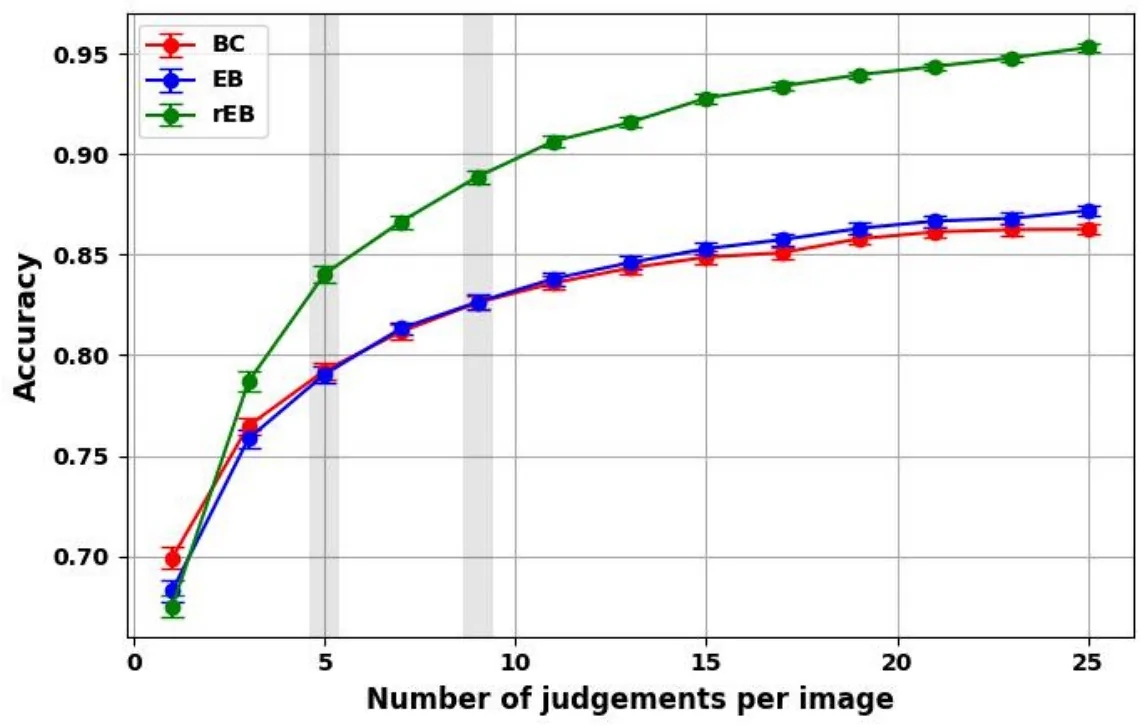
Crowd label accuracy as a function of the number of judgments per image in Experiment 2. The *error bars* represent the 95% confidence interval for the mean accuracy across the 100 simulations for each value of *N*. The *two gray bars* highlight the crowd-label accuracy at five and nine judgments per image because these datasets are used in the ML model training and evaluation

Next, we tested how crowd-label accuracy changes as a function of the number of judgments per image. This analysis was performed using the same procedure as that used in Experiment 1. Figure 13 depicts the impact of the number of judgments per image on accuracy. The EB and BC curves are almost identical, but the rEB curve lies well above both of them. Therefore, rEB labels reach higher overall accuracy and reach an arbitrary accuracy level with fewer judgments than EB and BC (industry-standard) labels.

**Fig. 14:**
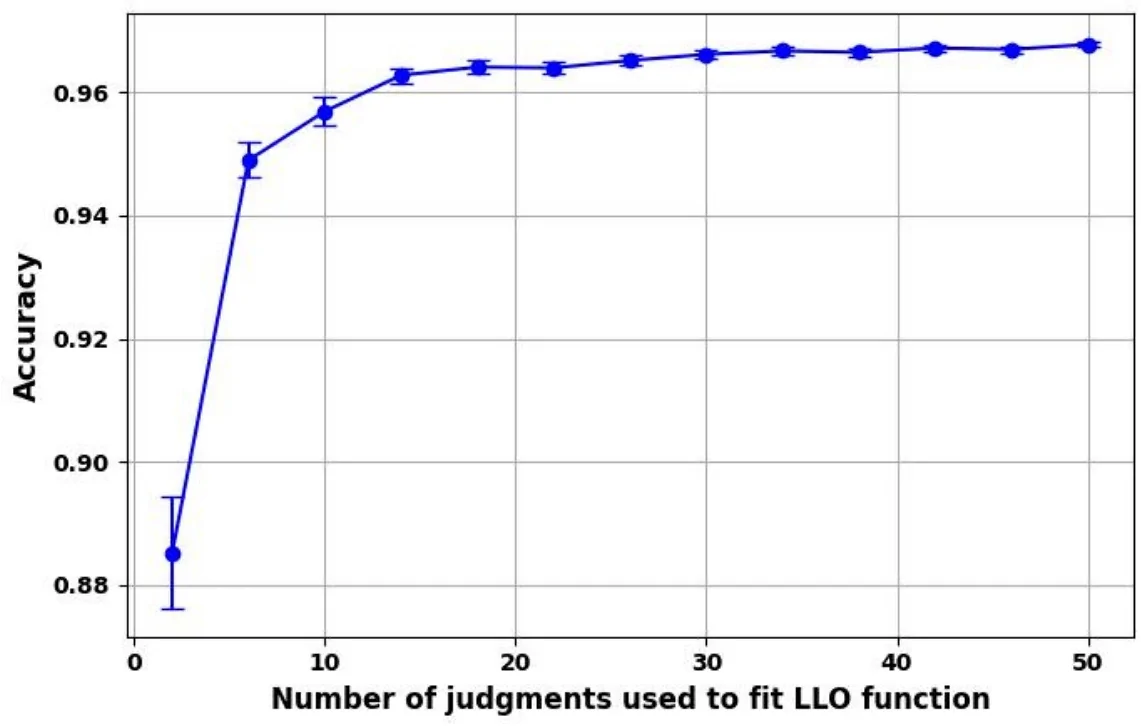
rEB crowd-label accuracy as a function of the number of judgments used to fit the LLO function in Experiment 2. The *error bars* represent the 95% confidence interval for the mean accuracy across the 100 simulations for each value of *N*

Additionally, we observed that the improved gains in accuracy as a result of recalibration in Experiment 2 (8.4 percentage point increase) exceeded that of Experiment 1 (3.5 percentage point). We believe the improved gains in accuracy are the result of participants having more accurate responses for the calibration set of images in Experiment 2 compared to that of Experiment 1. Participants were on average 62.9% accurate ($\sigma = 13.4\%$) at classifying images in the non-calibration set in Experiment 1 and were on average 69.5% accurate ($\sigma = 12.9\%$) at classifying images in the GS set in Experiment 2. To confirm this, we investigated how the number of judgments used to fit each participant’s LLO function impacted the rEB crowd accuracy in the QA set. This analysis was done using the same procedure as used in Experiment 1. Figure 14 illustrates the impact of the number of judgments in the calibration set on rEB crowd accuracy. Similar to Experiment 1, there are diminishing returns in terms of accuracy improvement with more than ten judgments per participant. The fact that almost all the gains in accuracy due to recalibration can be obtained using ten judgments or less suggests that the improved gain in accuracy in Experiment 2 compared to Experiment 1 is unlikely to be the result of having more judgments to fit the LLO function, but rather the result of having more accurate judgments to fit the LLO function.

### The effect of data re-calibration on CNN performance

We wanted to know whether differences in accuracy on the crowd-labeled datasets translated to the ML models trained on them. The analyses here follow those for Experiment 1. Results for model training can be found in Table 5 and results for model testing can be found in Table 6. Both tables show fairly similar trends to Experiment 1.

**Table 5:** Mean accuracy on the training folds with 95% confidence intervals for Experiment 2

Dataset	Number of judgments per image	Acc(crowd | true)	Acc(ML| crowd)	Acc(ML | true)
BC	5	.792 [.790, .794]	.925 [.921, .928]	.825 [.822, .828]
BC	9	.829 [.827, .830]	.963 [.960, .965]	.840 [.838, .843]
BC	all	.882 [.881, .882]	.993 [.993, .994]	.884 [.883, .885]
EB	5	.798 [.796, .799]	.927 [.924, .931]	.825 [.822, .829]
EB	9	.827 [.825, .828]	.961 [.958, .963]	.839 [.836, .842]
EB	all	.883 [.883, .884]	.989 [.988, .991]	.887 [.886, .888]
rEB	5	.839 [.838, .841]	.972 [.970, .974]	.858 [.856, .860]
rEB	9	.892 [.891, .894]	.989 [.988, .990]	.900 [.899, .902]
rEB	all	.967 [.966, .967]	.999 [.999, .999]	.967 [.966, .967]

**Table 6:** Mean accuracy on the testing folds with 95% confidence intervals for Experiment 2

Dataset	Number of judgments per image	Acc(crowd | true)	Acc(ML| crowd)	Acc(ML | true)
BC	5	.792 [.788, .796]	.770 [.765, .775]	.830 [.825, .836]
BC	9	.829 [.825, .832]	.815 [.811, .819]	.849 [.844, .854]
BC	all	.882 [.880, .884]	.907 [.904, .911]	.897 [.894, .900]
EB	5	.798 [.793, .802]	.777 [.773, .782]	.832 [.827, .837]
EB	9	.827 [.823, .830]	.811 [.807, .815]	.840 [.834, .845]
EB	all	.883 [.881, .885]	.896 [.894, .899]	.892 [.888, .896]
rEB	5	.839 [.836, .843]	.799 [.794, .803]	.895 [.892, .899]
rEB	9	.892 [.889, .895]	.864 [.860, .868]	.926 [.923, .929]
rEB	all	.967 [.966, .968]	.961 [.957, .964]	.960 [.958, .962]

First, Acc(crowd | true) increases as the number of judgments per image increases for BC, EB, and rEB datasets in both tables. For a fixed number of judgments per image, the accuracy of the rEB labels exceeded that of the EB and BC datasets, with the EB and BC datasets being roughly equal in terms of accuracy. All of these findings align with Fig. 13, which shows how accuracy increases as a function of the number of judgments per image, with rEB crowd labels being consistently more accurate than the EB and BC crowd labels.

Next, Acc(ML | crowd) increases as the number of judgments per image increases for BC, EB, and rEB datasets in both tables. Also, for a fixed number of judgments per image, the accuracy of the rEB labels almost always exceeds that of the EB and BC datasets, with the EB and BC datasets being roughly equal in terms of accuracy. Similar to Experiment 1, we see that Acc(ML | crowd) is much higher in the training folds compared to the testing folds for BC, EB, and rEB labels, which was expected given that we trained models on a relatively small dataset.

Lastly, Acc(ML | True) consistently increases as the number of judgments increases for BC, EB, and rEB datasets in both tables, and the accuracy of the rEB labels is greater than that of the EB and BC datasets for a fixed number of judgments. In fact, even with only five judgments per image, Acc(ML | True) for models trained on rEB labels matches that of the EB and BC datasets when using all judgments.

## Discussion

We use the term “cognitive-inspired data engineering” to describe a conceptual framework that integrates principles from cognitive science into the design and refinement of ML training data. This work serves as a proof of concept for this approach, investigating whether a model that corrects biases in human subjective probability judgments improves crowdsourced judgments for perceptual classification. Specifically, we evaluated the impact of recalibrating subjective probability judgments on the accuracy of crowdsourced medical image datasets and validated this approach by determining whether the improved accuracy propagated to the ML models trained on them. To do so, we collected judgments for a medical imaging task across two experiments: one where annotations were collected from a general crowdsourcing platform with novice annotators (MTurk) and another where annotations were collected from a more specialized crowdsourcing platform with skilled annotators (DiagnosUs). In both experiments, we used annotators’ judgments on a holdout set of images to recalibrate the remainder of their judgments. Our results were consistent across the two experiments in that recalibration via the LLO function led to more accurate labeled datasets and ML models.

First, in both experiments, we found that annotators tended to be overconfident in their judgments, favoring extreme judgments of 0% and 100% likelihood of blast. While recalibrating their judgments using the LLO function had little effect on individual annotator accuracy, it greatly improved the calibration of their judgments by transforming their response distribution to be more conservative. Furthermore, recalibration notably improved the accuracy of the resulting crowdsourced data.

Comparing the results across the two experiments, we found that the individual annotators were more accurate and better calibrated in Experiment 2 compared to that in Experiment 1, both before and after recalibration. The improved annotator performance in Experiment 2 also lead to more accurate crowdsourced datasets and models trained on these datasets compared to that in Experiment 1.

Domains such as medical image diagnostics have begun to turn to crowdsourcing as a viable method to annotate unlabeled data because of the speed and cost-efficiency offered by crowdsourcing (Eickhoff et al., 2012). These two objectives can be further optimized by reducing the number of annotations per stimulus without degrading the quality of the dataset. In both experiments, we found that fewer rEB judgments are required, compared to EB and BC judgments, to produce a dataset at an arbitrary level of accuracy. While recalibration does require an additional set of judgments on a holdout set of images used solely for the purposes of recalibration, we found that most of the gains in accuracy due to recalibration can be achieved with as few as six judgments on images from the holdout set. Therefore, beyond just improving the accuracy of the crowdsourced labels at a given number of judgments per image, recalibrated crowdsourced datasets achieve an arbitrary level of accuracy at a fraction of the number of annotations compared to uncalibrated crowdsourced datasets, which can greatly reduce the time and cost spent on annotating data. This improved annotation efficiency can accelerate the creation of large, labeled datasets, addressing the present-day, primary bottleneck in the development of medical AI (Ørting et al., 2019).

### Relation to previous work

Turner et al. (2014) also used the LLO function to recalibrate individuals’ judgments and studied the impact of aggregated judgments on forecasting accuracy. Unlike in our work, Turner et al. (2014) included a model variant which constrained the parameters of the LLO function using a hierarchical distribution when fitting to each annotator because they had few judgments from some annotators. We did not find this additional step necessary since we collected several judgments per annotator to fit the LLO function. Moreover, we found that fitting the LLO function with very few judgments led to gains in crowdsourced dataset accuracy. Our study also differs from Turner et al. (2014) in that we studied the impact of recalibration in the context of crowdsourcing ML training datasets for a complex perceptual task, whereas Turner et al. (2014) used recalibrated judgments on 176 forecasting questions (e.g., regarding Greek debt default and US credit rating). Despite the different goals of our work and Turner et al. (2014), we find it promising that recalibration is successful at improving accuracy across the disparate domains of forecasting and medical image classification. This suggests recalibration is a general technique likely to succeed in other domains.

Epping et al. (2026) extend this approach in a related but distinct direction by asking how recalibrated judgments can be used to train AI decision aids that are not only accurate, but also calibrated and aligned with expert uncertainty. Whereas the present work focuses on whether individual-level recalibration improves the accuracy and efficiency of crowdsourced datasets and whether those improvements propagate to ML models trained on binarized crowd labels, Epping et al. (2026) examine additional stages of the data pipeline. Specifically, they evaluate crowd-level recalibration and compare models trained on hard versus soft labels, where soft labels preserve uncertainty in the aggregated judgments. They find that individual-level recalibration improves dataset and model accuracy, that crowd-level recalibration further improves accuracy for BC and EB datasets while substantially improving calibration across all dataset types, and that models trained on recalibrated soft labels show stronger alignment with expert judgments, although this can come at the cost of poorer model calibration. Thus, the two papers are complementary but distinct: the present paper establishes the value of recalibrating individual annotator judgments for improving crowdsourced training data and downstream model accuracy, whereas Epping et al. (2026) examine how recalibration interacts with soft-label training and expert-model alignment in the broader context of AI-augmented decision-making.

### Limitations

Because our primary goal was to investigate the impact of recalibration on crowdsourced dataset and model accuracy, we limited ourselves to simple algorithms for aggregating individual annotations into crowdsourced labels. That is, for the BC dataset, the crowd label for a given image was the label with the most annotations for that image and, for the EB and rEB datasets, the crowd label was the mean judgment across annotations for that image. More sophisticated aggregation algorithms involve taking each annotator’s past performance into account, such as only using annotations from the most accurate annotators (Budescu & Chen, 2015; Galesic et al., 2018; Himmelstein et al., 2023) or weighting judgments based on a annotator’s performance (Armstrong, 2001; Atanasov et al., 2017; Collins et al., 2023; Duhaime et al., 2023; Wang et al., 2011). While we chose to simplify our aggregation algorithms to focus on the impact of recalibration, we believe recalibrating judgments using the LLO function offers several advantages over the previously mentioned aggregation algorithms.

First, weighting judgments based on annotator performance requires a large number of judgments on labeled images and a decision regarding how to convert past performance into a weight (Collins et al., 2023). For example, annotators could be weighted using moving-average accuracy, but the number of judgments included in the moving average is an additional decision. Second, using only annotations from the most accurate annotators increases the total number of judgments that must be collected. For example, even if the same level of accuracy can be achieved using five recalibrated judgments per image as using five high-quality, uncalibrated judgments per image, and high-quality judgments are only sampled from the more accurate half of annotators, then one would still need to collect ten judgments per image overall. Plus, the threshold used to determine whether an annotator’s judgments should be included in the crowd-label computation needs to be determined and will likely differ on a case-by-case basis. Finally, more sophisticated algorithms introduce complexity, which adds another layer of difficulty to curating labeled datasets, for example, see Hasan et al. (2022); Wilson and Farrow (2018); Soule et al. (2024). Altogether, recalibration using the LLO function is a simple transformation that can be applied to any task involving crowdsourced subjective probability judgments and this transformation does not involve additional decisions and is very label-efficient in that high levels of accuracy can be achieved without filtering out labels from less capable annotators. Note that the recalibration technique outlined here does not preclude any of the other accuracy-based approaches and therefore can be combined with them to potentially improve the quality of crowdsourced datasets further.

Another limitation is that we only used one set of stimuli in our study. We used the same dataset in both experiments so we could more easily test whether our results generalize from the lab to the field. However, the recalibration mechanism we employ is grounded in a broader literature demonstrating that recalibration can improve probabilistic judgments across diverse non-medical domains, including geopolitical and economic forecasting (Han & Budescu, 2022; Turner et al., 2014). While future work should directly test the present approach on additional tasks and datasets, these findings support broader applicability beyond medical imaging.

Finally, we note two study design choices that limit the scope of our work. First, blast and nonblast images were sampled evenly in each of our studies, which allowed us to study the impact of recalibration without annotator judgments being influenced by prevalence effects (Trueblood et al., 2021; Wolfe et al., 2007). In situations where there are substantial class imbalances, individuals often show increased error rates. For example, in the case of low prevalence (e.g., blast cells are very rare), miss rates are elevated as compared to a situation with equal prevalence. Future work should investigate whether recalibration approaches can correct prevalence-induced errors. Second, when training ML models, we binarized crowd judgments, as training models on discrete class labels is one of the most common approaches in supervised ML. Training models directly on continuous probability estimates rather than binarized ones may better preserve the additional information contained in subjective judgments that could be used to further improve model quality (de Vries & Thierens, 2025; Peterson et al., 2019). Additionally, there is growing evidence that human-AI decision-making improves when AI systems provide probabilities that reflect human-like uncertainty (Corvelo Benz & Rodriguez, 2023; Tschandl et al., 2020). As discussed above, Epping et al. (2026) begins to address this question; understanding how recalibration interacts with varying base rates and alternative training approaches more broadly remains a promising direction for future work.

## Conclusion

Supervised ML models are designed to learn statistical regularities in their training data and therefore are likely to inherit annotator biases embedded in their training dataset. Many techniques to correct these biases in ML models have approached the problem from a model perspective (adjusting the model algorithm or objective function) rather than from a data perspective, which is unsurprising given that model engineering has received far more attention compared to data engineering (Sambasivan et al., 2021; Wagstaff, 2012). Cognitive-inspired data engineering approaches the problem from the data perspective, focusing specifically on the cognitive biases that pervade annotators’ decision-making processes. Cognitive scientists have long studied cognitive biases in human judgment and decision-making and a better understanding of these biases can assist in mitigating their presence in ML training datasets. The primary contribution of this work is a cognitively grounded approach to correcting biases at the annotation level. By improving the quality of crowdsourced labels, this approach yields consistent downstream benefits for machine learning models without requiring changes to model architectures or training procedures.

## Supplementary Information

Below is the link to the electronic supplementary material.

Supplementary file 1 (pdf 409 KB)

## Data Availability

Data used to produce the results in this article and materials are available on OSF at https://osf.io/y3j82/?view_only=9be0a79e323a4009bdcaf7af9eb3f917. We can provide the white blood cell images upon request. The experimental procedure, sample size, and exclusion criteria for Experiment 1 were pre-registered on AsPredicted (#135800, https://aspredicted.org/zgq6-8y6s.pdf). The hypotheses and proposed analysis in the pre-registration focused on how recalibrating crowd labels affects the expected calibration error of crowd labels and ML models trained on soft crowd labels. We did not anticipate that individual-level recalibration would be as successful, given the relatively small number of judgments used to fit the recalibration model. When we found that individual-level recalibration had a strong, positive impact on crowd-label accuracy, we decided to focus this article on how individual-level recalibration affects the accuracy of crowd labels and ML models trained on them. The original proposed analysis outlined in the pre-registration will be published in a separate journal article. Experiment 2 was not pre-registered.

## Code availability

All code used to produce the results in this article are available on OSF at https://osf.io/y3j82/?view_only=9be0a79e323a4009bdcaf7af9eb3f917.
