# Supplementary material for: Improving crowdsourcing for AI through cognitive-inspired data engineering
Source: Behav Res Methods. 2026 Sep 1;58(10):283. doi: 10.3758/s13428-026-03160-4 (PMC13534169; doi:10.3758/s13428-026-03160-4)
Supplement: Supplementary file 1 [file 13428_2026_3160_MOESM1_ESM.pdf]

1 EXPERIMENT 1

1.1 CNN hyperparameter grid search results

Number of training epochs	Learning rate	Minibatch size	L2-regularization strength	EB 5 votes per image	EB 9 votes per image	EB all votes per image	rEB 5 votes per image	rEB 9 votes per image	rEB all votes per image
5	1e-4	16	1e-3	.5584	.4754	.3070	.4736	.4136	.2446
5	1e-4	16	5e-3	.5556	.4753	.3078	.4734	.4128	.2444
5	1e-4	16	1e-2	.5552	.4737	.3066	.4706	.4124	.2462
5	1e-4	24	1e-3	.5479	.4589	.3088	.4726	.4088	.2459
5	1e-4	24	5e-3	.5455	.4589	.3091	.4724	.4095	.2469
5	1e-4	24	1e-2	.5440	.4575	.3097	.4710	.4089	.2482
5	1e-4	32	1e-3	.5414	.4576	.3165	.4735	.4158	.2598
5	1e-4	32	5e-3	.5398	.4572	.3175	.4721	.4166	.2603
5	1e-4	32	1e-2	.5369	.4558	.3184	.4706	.4147	.2622
5	5e-4	16	1e-3	.5757	.4859	.3303	.4959	.4328	.2523
5	5e-4	16	5e-3	.5607	.4889	.3268	.4943	.4189	.2357
5	5e-4	16	1e-2	.5505	.4842	.3216	.4923	.4203	.2452
5	5e-4	24	1e-3	.5988	.5159	.3369	.5110	.4474	.2640
5	5e-4	24	5e-3	.5858	.4985	.3238	.4919	.4467	.2602
5	5e-4	24	1e-2	.5758	.4870	.3227	.4881	.4318	.2660
5	5e-4	32	1e-3	.6028	.5143	.3351	.5300	.4463	.2649
5	5e-4	32	5e-3	.5936	.5018	.3207	.5184	.4502	.2511
5	5e-4	32	1e-2	.5804	.4972	.3288	.5087	.4363	.2597
5	1e-3	16	1e-3	.5592	.4940	.3489	.5015	.4396	.2717
5	1e-3	16	5e-3	.5530	.4897	.3609	.4965	.4364	.2711
5	1e-3	16	1e-2	.5786	.5104	.3730	.5227	.4539	.3217
5	1e-3	24	1e-3	.5769	.5114	.3413	.4911	.4344	.2566
5	1e-3	24	5e-3	.5604	.4790	.3517	.4764	.4332	.2597
5	1e-3	24	1e-2	.5590	.4801	.3663	.5188	.4257	.2782
5	1e-3	32	1e-3	.5769	.5350	.3624	.4989	.4459	.2523
5	1e-3	32	5e-3	.5498	.4891	.3364	.4897	.4364	.2696
5	1e-3	32	1e-2	.5625	.4943	.3380	.5031	.4251	.2640
10	1e-4	16	1e-3	.6886	.5716	.3401	.5842	.4980	.2717
10	1e-4	16	5e-3	.6883	.5686	.3420	.5837	.4966	.2695
10	1e-4	16	1e-2	.6929	.5638	.3332	.5730	.4923	.2695
10	1e-4	24	1e-3	.6822	.5662	.3334	.5663	.4946	.2638
10	1e-4	24	5e-3	.6791	.5539	.3332	.5616	.4931	.2599
10	1e-4	24	1e-2	.6699	.5515	.3260	.5611	.4876	.2594
10	1e-4	32	1e-3	.6690	.5333	.3163	.5507	.4727	.2608
10	1e-4	32	5e-3	.6607	.5291	.3174	.5482	.4688	.2609
10	1e-4	32	1e-2	.6482	.5225	.3176	.5429	.4635	.2603
10	5e-4	16	1e-3	.6174	.5363	.3404	.5578	.4726	.2685
10	5e-4	16	5e-3	.5728	.5085	.3181	.5248	.4303	.2554
10	5e-4	16	1e-2	.5575	.4993	.3306	.5059	.4281	.2592
10	5e-4	24	1e-3	.6501	.5591	.3503	.5943	.5012	.2948
10	5e-4	24	5e-3	.6207	.5255	.3186	.5544	.4746	.2752
10	5e-4	24	1e-2	.5820	.4977	.3412	.5340	.4505	.2566

10	5e-4	32	1e-3	.6794	.5822	.3760	.6086	.5006	.2747
10	5e-4	32	5e-3	.6397	.5450	.3463	.5541	.4725	.2619
10	5e-4	32	1e-2	.6109	.5243	.3379	.5611	.4600	.2622
10	1e-3	16	1e-3	.5418	.5173	.3398	.5021	.4716	.2650
10	1e-3	16	5e-3	.5766	.5578	.3851	.5291	.4495	.2956
10	1e-3	16	1e-2	.6310	.5682	.4783	.5783	.5630	.3976
10	1e-3	24	1e-3	.5895	.5103	.3609	.5213	.4891	.2757
10	1e-3	24	5e-3	.5709	.5131	.3770	.5171	.4548	.2924
10	1e-3	24	1e-2	.5995	.5368	.4071	.5404	.5007	.3595
10	1e-3	32	1e-3	.6146	.5163	.3493	.5430	.4567	.2657
10	1e-3	32	5e-3	.5873	.4903	.3490	.5099	.4470	.2857
10	1e-3	32	1e-2	.5642	.5429	.3980	.5334	.4604	.3500
15	1e-4	16	1e-3	.8351	.6778	.3739	.6944	.5825	.2996
15	1e-4	16	5e-3	.8228	.6786	.3733	.6855	.5746	.2879
15	1e-4	16	1e-2	.8081	.6585	.3738	.6610	.5675	.2905
15	1e-4	24	1e-3	.8195	.6851	.3861	.7007	.5845	.2885
15	1e-4	24	5e-3	.8154	.6668	.3793	.6824	.5764	.2877
15	1e-4	24	1e-2	.8046	.6537	.3706	.6818	.5567	.2783
15	1e-4	32	1e-3	.8272	.6670	.3692	.6645	.5581	.2980
15	1e-4	32	5e-3	.8138	.6548	.3716	.6571	.5534	.2936
15	1e-4	32	1e-2	.7814	.6377	.3661	.6441	.5448	.2915
15	5e-4	16	1e-3	.6590	.5774	.3395	.6027	.5284	.2739
15	5e-4	16	5e-3	.5941	.5038	.3385	.5075	.4483	.2628
15	5e-4	16	1e-2	.5749	.5064	.3396	.5131	.4478	.2675
15	5e-4	24	1e-3	.6783	.6183	.3802	.6421	.5279	.2902
15	5e-4	24	5e-3	.6296	.5342	.3318	.5707	.4987	.2611
15	5e-4	24	1e-2	.5988	.4948	.3287	.5326	.4477	.2598
15	5e-4	32	1e-3	.7586	.6464	.3728	.6574	.5591	.2870
15	5e-4	32	5e-3	.6678	.5570	.3507	.5755	.4960	.2657
15	5e-4	32	1e-2	.6264	.5327	.3206	.5436	.4427	.2579
15	1e-3	16	1e-3	.6079	.5191	.3300	.5309	.4566	.2689
15	1e-3	16	5e-3	.5834	.5484	.3914	.5274	.4665	.3459
15	1e-3	16	1e-2	.5900	.5714	.4261	.5835	.5074	.3818
15	1e-3	24	1e-3	.6149	.5230	.3515	.5394	.4831	.2804
15	1e-3	24	5e-3	.6116	.5153	.3704	.5510	.4671	.2938
15	1e-3	24	1e-2	.6323	.5440	.4354	.5722	.5098	.3639
15	1e-3	32	1e-3	.6350	.5523	.3576	.5480	.4711	.2826
15	1e-3	32	5e-3	.5991	.5007	.3633	.5359	.4730	.2955
15	1e-3	32	1e-2	.5836	.5635	.4195	.5962	.5065	.3626

SUPPLEMENTARY TABLE 1. Hyperparmeter grid search results for Experiment 1. The 6 leftmost columns are the mean loss (the average binary cross entropy loss between the machine output and the crowd labels) averaged across the 30 training iterations for each of the training datasets in Experiment 1. The highlighted cells are the lowest mean loss for the entire column and therefore refer to the hyperparameter combination used for that dataset in the main text.

1.2 Individual accuracy distribution in each condition

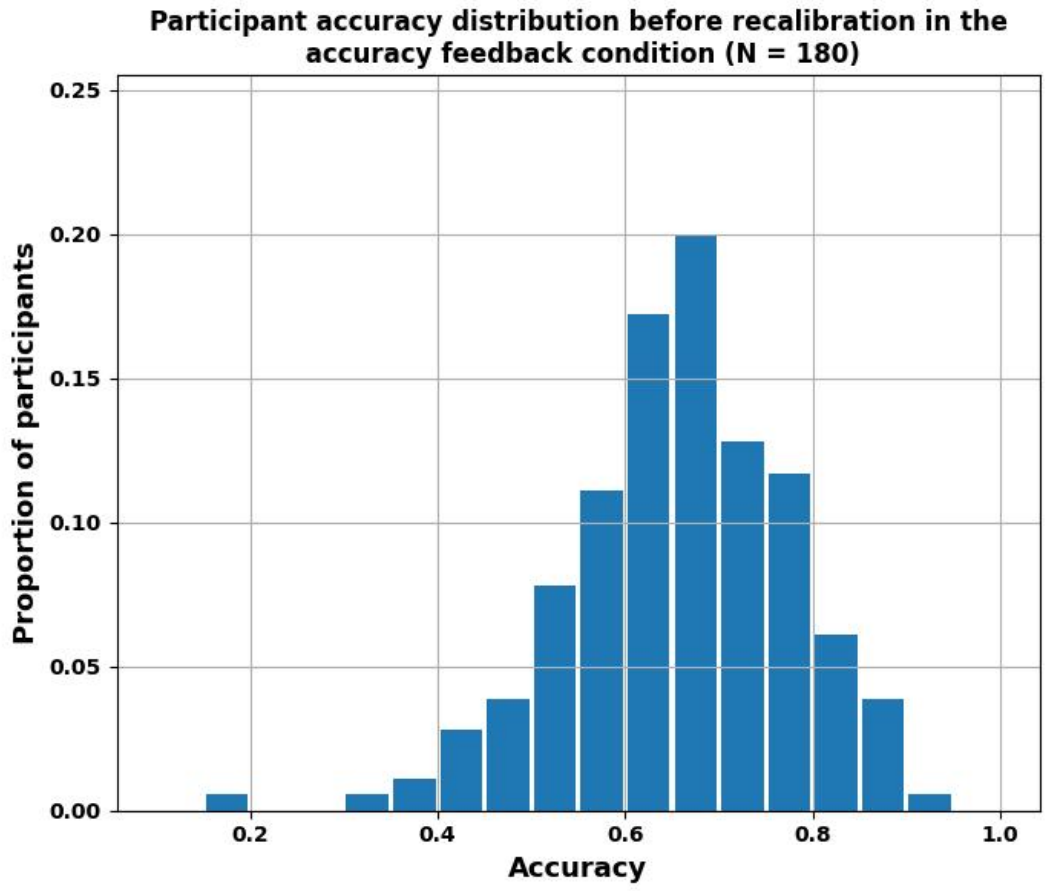

(a) Participant accuracy distribution before their judgments were calibrated using the LLO function in the accuracy feedback condition.

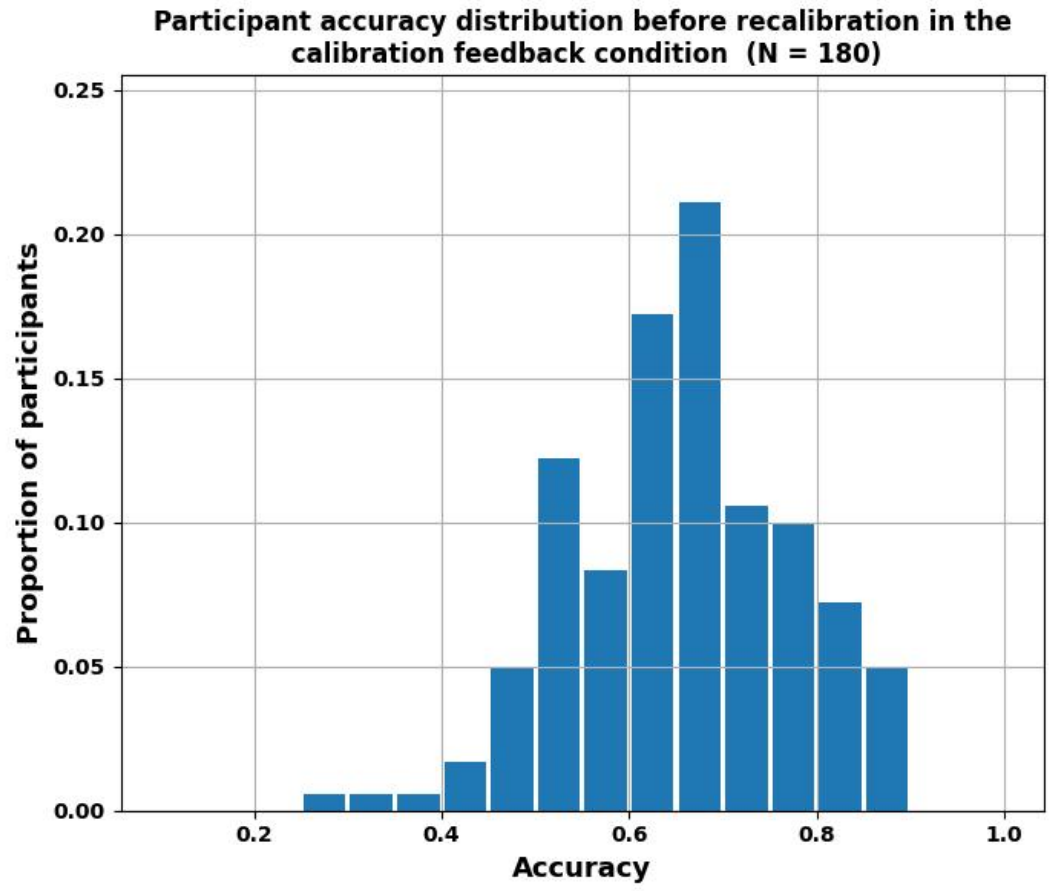

(b) Participant accuracy distribution before their judgments were calibrated using the LLO function in the calibration feedback condition.

SUPPLEMENTARY FIGURE 1. Participant accuracy distribution on images in the non-calibration set for the two conditions in Experiment 1.

1.3 | Impact of fitting LLO function with 2, 6, and 10 judgments

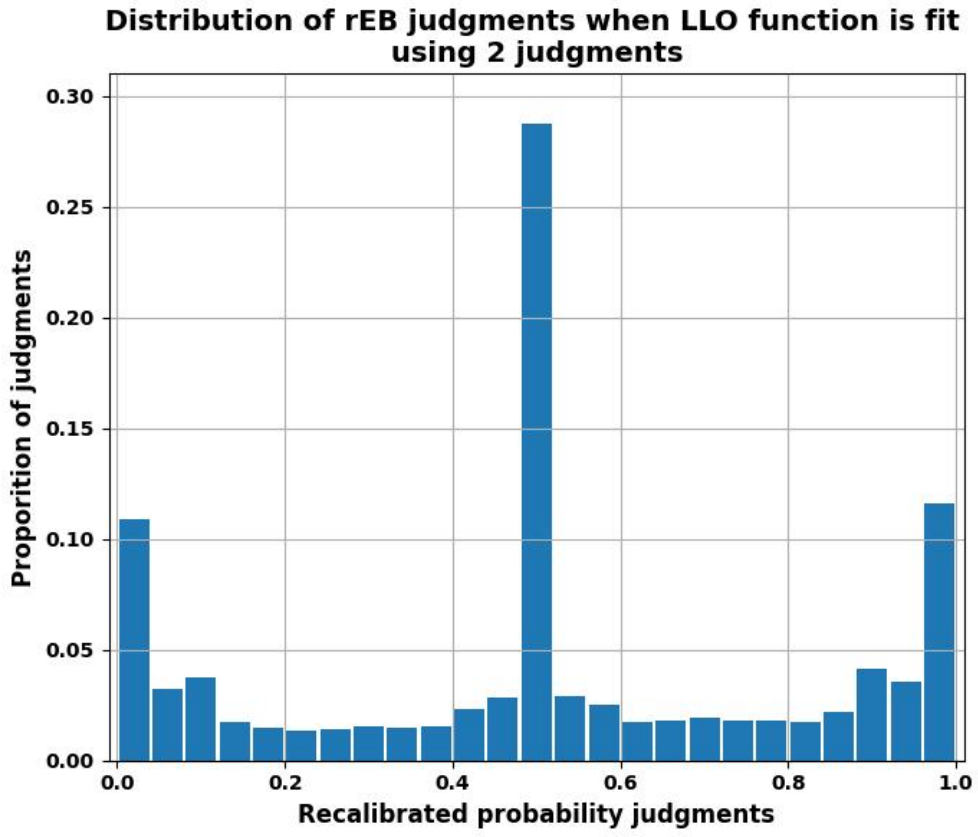

(a) Participant response distribution after their responses were calibrated using the LLO function based on 2 judgments.

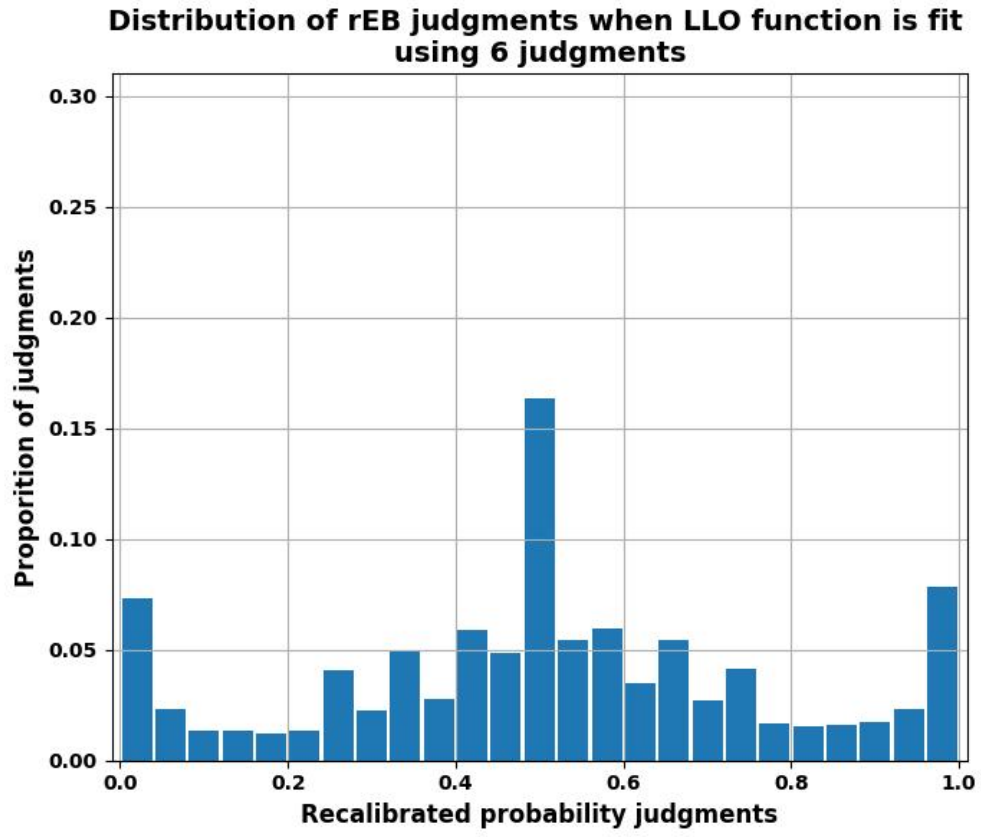

(b) Participant response distribution after their responses were calibrated using the LLO function based on 6 judgments.

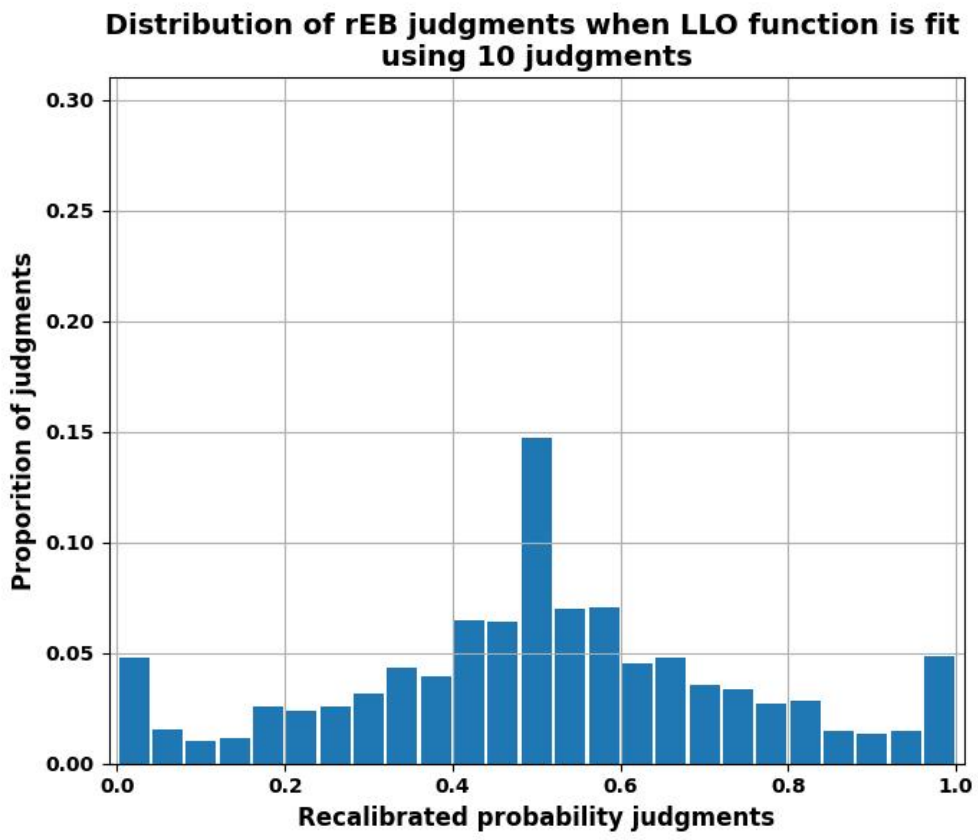

(c) Participant response distribution after their responses were calibrated using the LLO function based on 10 judgments.

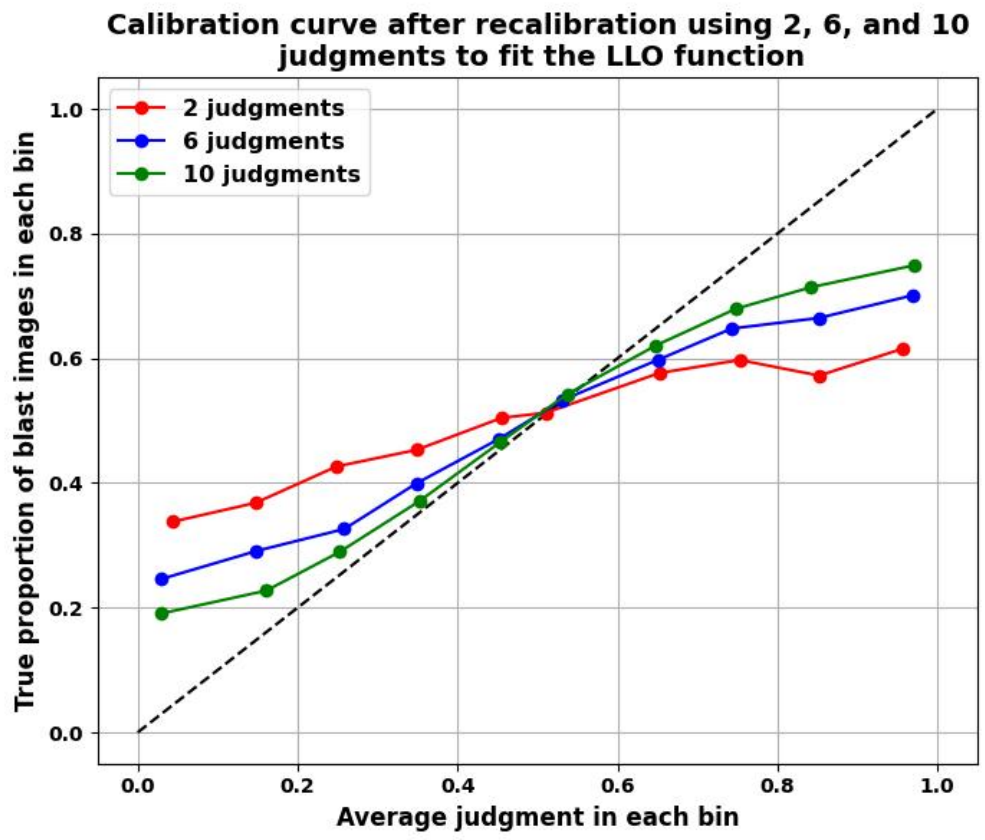

(d) Calibration curve after participants' responses were calibrated using the LLO function based on 2, 6, and 10 judgments.

SUPPLEMENTARY FIGURE 2. Participant response distribution and calibration curves after their responses were calibrated using the LLO function based on 2, 6, and 10 judgments in Experiment 1.

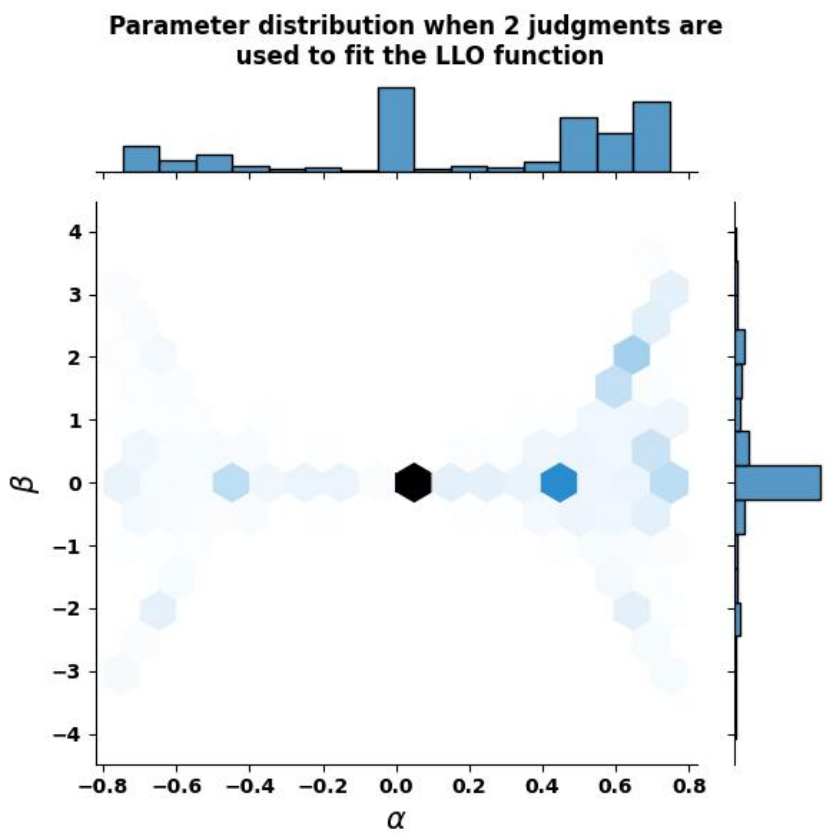

(a) Best fit α and β parameters for the LLO when fit using 2 judgments for participants in Experiment 1.

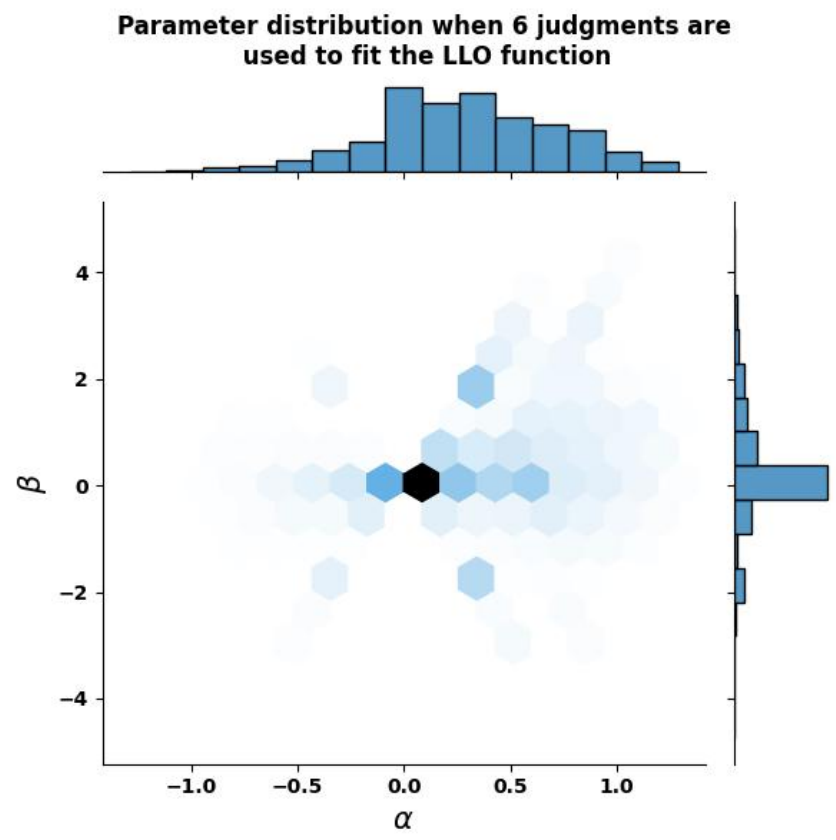

(b) Best fit α and β parameters for the LLO when fit using 6 judgments for participants in Experiment 1.

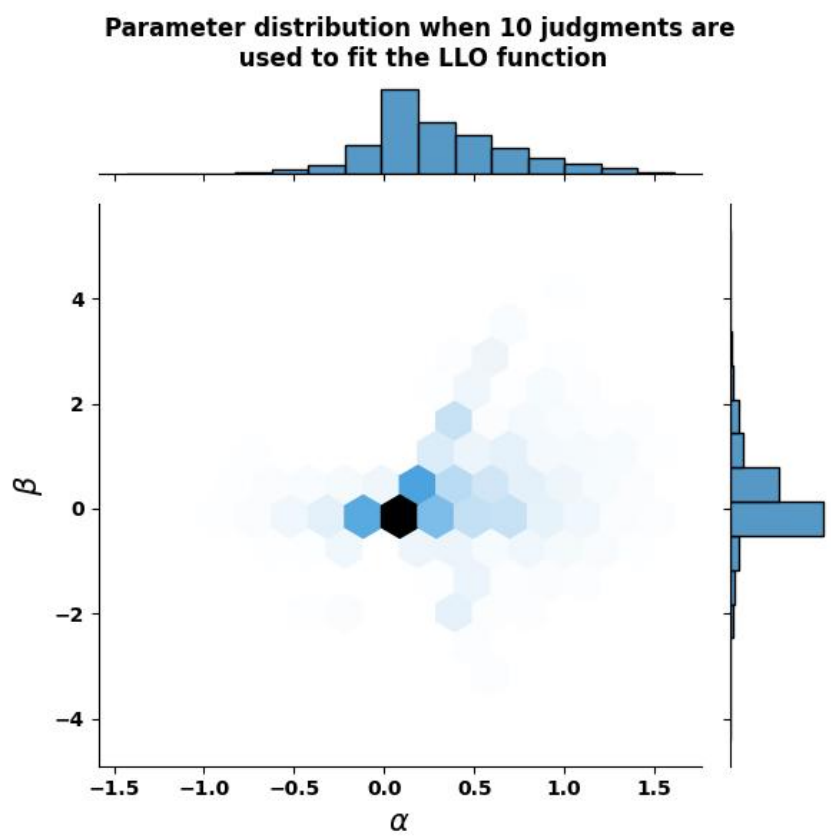

(c) Best fit α and β parameters for the LLO when fit using 10 judgments for participants in Experiment 1.

SUPPLEMENTARY FIGURE 3. Best fit parameters for the LLO function when fit using 2, 6, and 10 judgments for participants in Experiment 1.

2 | EXPERIMENT 2

2.1 | Distribution of difficulty ratings

Out of the 549 image, there were 300 images in the QA set and 249 images in the GS set. Out of the 300 images in the QA set, 150 were blast images and 150 were nonblast images. Out of the 249 images in the GS set, 116 were blast images and 133 were nonblast images. Table 2 indicates the number of images in each difficulty category, partitioned by the image class (blast or nonblast) and the image set (QA or GS).

Image class	Image set	Very easy	Easy	Medium	Hard	Very hard
Blast	QA	22	39	38	33	18
Blast	GS	17	30	30	26	13
Nonblast	QA	32	36	26	22	34
Nonblast	GS	29	31	23	19	31

SUPPLEMENTARY TABLE 2. Difficulty distribution for image in Experiment 2, partitioned by the image class and image set. The numbers in the 5 difficult categories correspond to the number of images in each category, given the image class and image set.

2.2 | Task instructions

Task instructions were split across multiple slides. Below are the instructions on each slide for each condition.

2.2.1 | EB condition

2.2.1.1 | *Slide 1*

How is performance evaluated in this contest? Unlike other competitions on DiagnosUs, you will indicate the likelihood (from 0% to 100%) that you believe the case is a blast cell. Your score is based on how far away your response is from the correct answer. When your response is quite a bit off from the correct answer, you'll lose more points, which really emphasizes the importance of making careful guesses. You will have the opportunity to learn how your responses are turned into scores during the practice cases.

2.2.1.2 | *Slide 2*

Some quick background: this is a blast cell, a pathological white blood cell whose presence is often a sign of blood cancers such as leukemia.

2.2.1.3 | *Slide 3*

Half of the images will contain a blast cell and half will contain a non-blast cell instead. A non-blast cell is a different type of white blood cell that is not linked to blood cancers.

2.2.1.4 | *Slide 4*

In day to day life, we often think about how likely something is, such as the probability of rain tomorrow, the probability that housing prices will go up, and for health professionals, the probability a white blood cell is a blast cell.

In this task, you might think a cell is 70% likely to be a blast cell. You could answer with any number from 0% to 100%. Suppose you answer 100% blast, trying to get the highest score. In this scenario, your true belief would suggest you have a 70% chance to earn a score of 100 in the case that the cell is actually a blast cell. However, you also have a 30% chance to earn a score of 0, in the case that the lesion is not a blast cell! You would be better off answering with your true belief of 70%, which will maximize the score you can expect. For each case, you will give yourself the highest expected score by truthfully telling us what you believe.

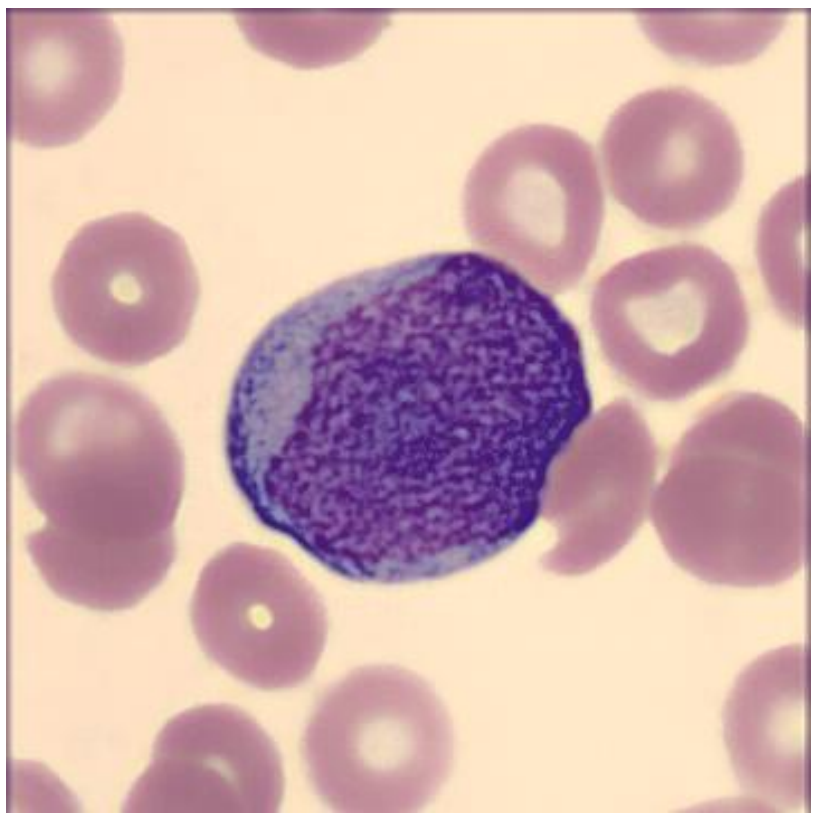

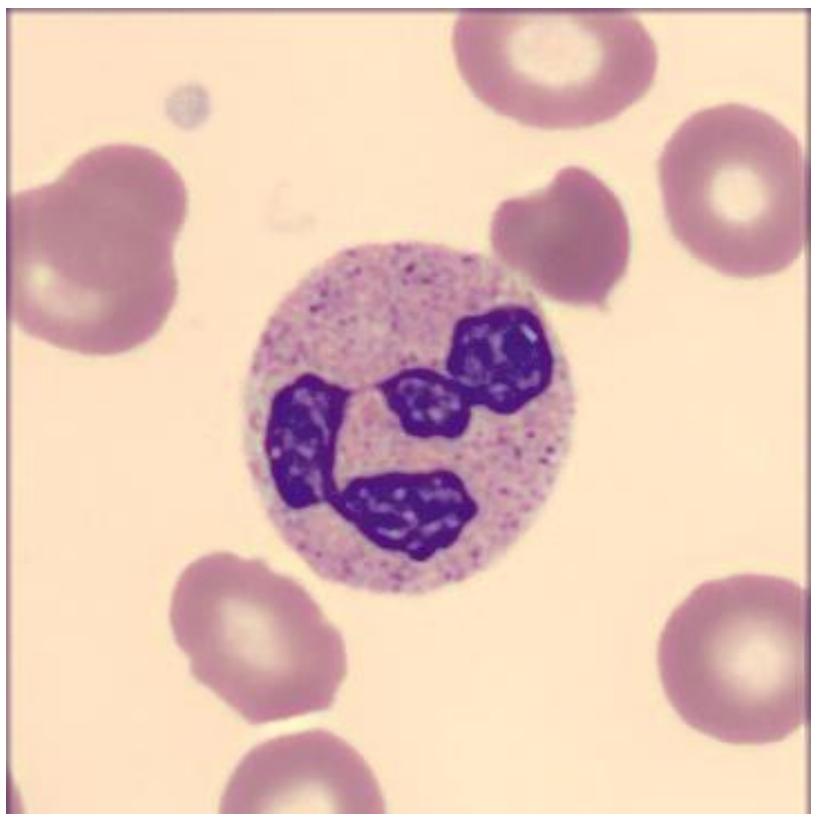

2.2.1.5 | *Slide 5*

Remember: Think carefully about the likelihood that each case is a blast cell and report the number that reflects your true belief. For example, unless you are absolutely sure that an image contains a blast cell, you would be better off responding with something less than 100% instead of 100%.

In previous contests, people who mostly responded they were completely certain (a likelihood of 0% or 100%) were less likely to make the leaderboard.

Good luck!

2.2.2 | BC condition

2.2.2.1 | *Slide 1*

How is performance evaluated in this contest? You will indicate whether or not you believe the case is a blast cell. Your score is based on whether your response is the correct answer. You will have the opportunity to learn how your responses are turned into scores during the practice cases.

2.2.2.2 | *Slide 2*

Some quick background: this is a blast cell, a pathological white blood cell whose presence is often a sign of blood cancers such as leukemia.

2.2.2.3 | *Slide 3*

Half of the images will contain a blast cell and half of the images will contain a non-blast cell instead. A non-blast cell is a different type of white blood cell that is not linked to blood cancers.

2.2.2.4 | *Slide 4*

Good luck!

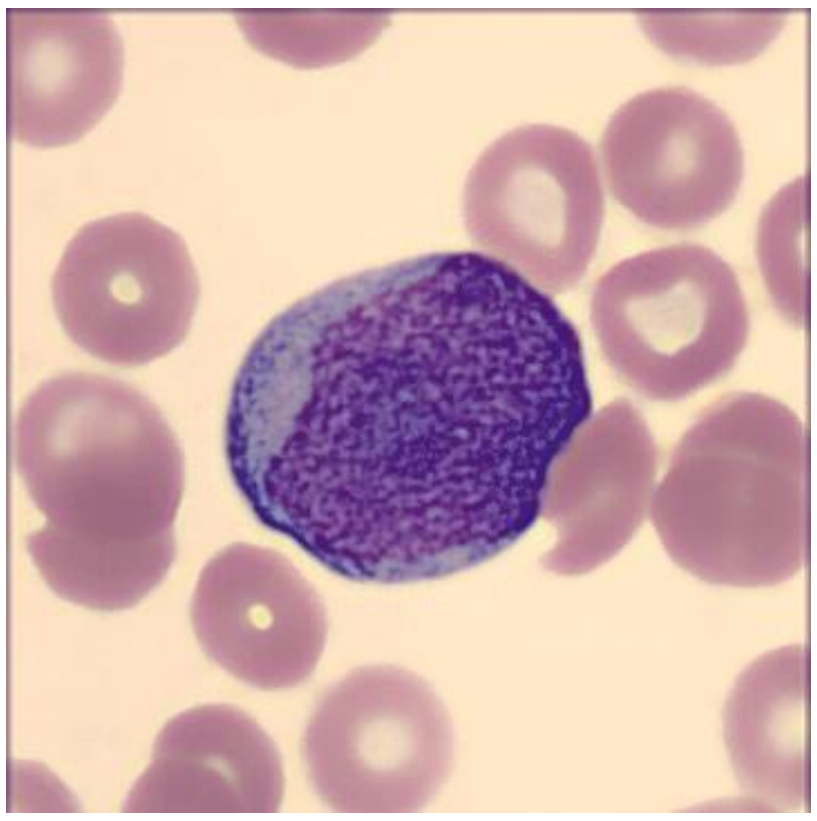

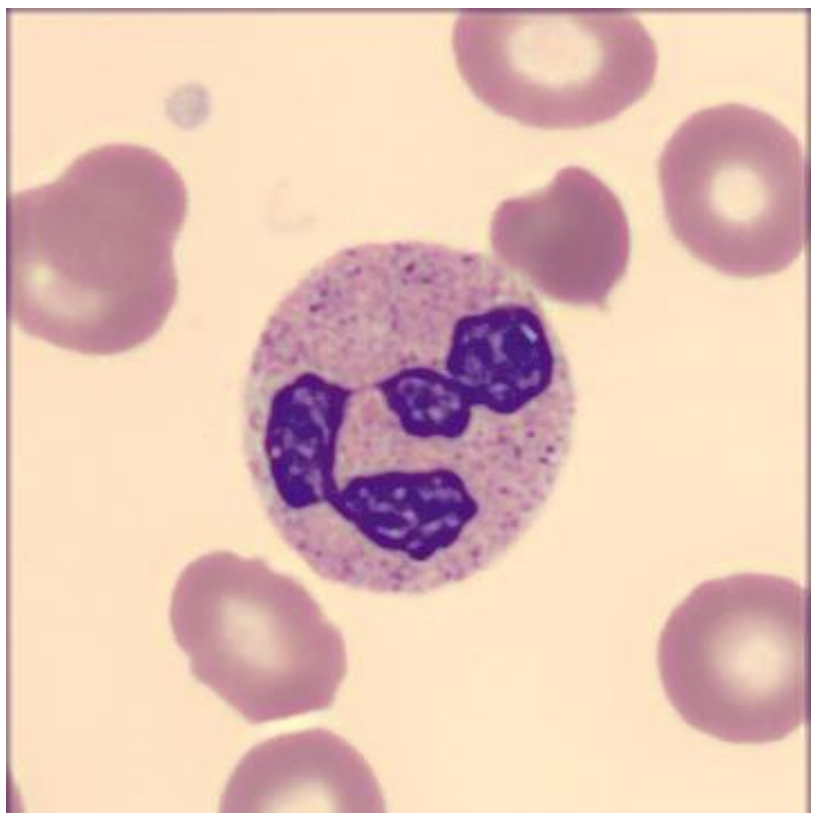

2.3 | CNN hyperparameter grid search results

Number of training epochs	Learning rate	Minibatch size	L2-regularization strength	BC 5 votes per image	BC 9 votes per image	BC all votes per image	EB 5 votes per image	EB 9 votes per image	EB all votes per image	rEB 5 votes per image	rEB 9 votes per image	rEB all votes per image
5	1e-4	16	1e-3	.5036	.4278	.2723	.4770	.4149	.2503	.5106	.3712	.1356
5	1e-4	16	5e-3	.5016	.4260	.2730	.4751	.4136	.2492	.5092	.3696	.1371
5	1e-4	16	1e-2	.5023	.4238	.2724	.4744	.4124	.2499	.5068	.3698	.1381
5	1e-4	24	1e-3	.5121	.4323	.3207	.4875	.4240	.2789	.5038	.3802	.1605
5	1e-4	24	5e-3	.5106	.4304	.3199	.4864	.4234	.2786	.5046	.3809	.1643
5	1e-4	24	1e-2	.5093	.4288	.3212	.4854	.4228	.2822	.5046	.3830	.1689
5	1e-4	32	1e-3	.5220	.4411	.3551	.5082	.4393	.3207	.5112	.3950	.1946
5	1e-4	32	5e-3	.5220	.4398	.3544	.5082	.4392	.3217	.5113	.3968	.1996
5	1e-4	32	1e-2	.5224	.4403	.3559	.5051	.4390	.3230	.5116	.3989	.2063
5	5e-4	16	1e-3	.5853	.4830	.2870	.5237	.4540	.2945	.5666	.4276	.1777
5	5e-4	16	5e-3	.5663	.4674	.2824	.5106	.4498	.2789	.5612	.4039	.1763
5	5e-4	16	1e-2	.5621	.4801	.2622	.5163	.4424	.2678	.5516	.4029	.1901
5	5e-4	24	1e-3	.6201	.4828	.3076	.5553	.4869	.2814	.6001	.4081	.1718
5	5e-4	24	5e-3	.5907	.4663	.2935	.5309	.4711	.2850	.5812	.4056	.1726
5	5e-4	24	1e-2	.5814	.4486	.2839	.5234	.4513	.2865	.5834	.3918	.1671
5	5e-4	32	1e-3	.6225	.4954	.3367	.5792	.5054	.3348	.6156	.4500	.2246
5	5e-4	32	5e-3	.6012	.4959	.3090	.5555	.4754	.3230	.6045	.4227	.1964
5	5e-4	32	1e-2	.5856	.4661	.2957	.5506	.4650	.3288	.6032	.4178	.1823

5	1e-3	16	1e-3	.5855	.5203	.3264	.5402	.4801	.2950	.5705	.4390	.2372
5	1e-3	16	5e-3	.5423	.4376	.3184	.5069	.4404	.2748	.5473	.4258	.1962
5	1e-3	16	1e-2	.5225	.4537	.3147	.5156	.4541	.3007	.5464	.4210	.2188
5	1e-3	24	1e-3	.6367	.5213	.3567	.5626	.4707	.3423	.6202	.4411	.2553
5	1e-3	24	5e-3	.5670	.5017	.3102	.5376	.4444	.2970	.6054	.4093	.2559
5	1e-3	24	1e-2	.5524	.4775	.2946	.4981	.4260	.3041	.5656	.4107	.2040
5	1e-3	32	1e-3	.6371	.5520	.3774	.6066	.5374	.3435	.6483	.4795	.2891
5	1e-3	32	5e-3	.5687	.5268	.3309	.5571	.4724	.3164	.5687	.4458	.2588
5	1e-3	32	1e-2	.5397	.4916	.3118	.5365	.4620	.3041	.5628	.4191	.2049
10	1e-4	16	1e-3	.6289	.5191	.2832	.6070	.5140	.2605	.6235	.4437	.1276
10	1e-4	16	5e-3	.6176	.5207	.2783	.5993	.5099	.2596	.6197	.4362	.1283
10	1e-4	16	1e-2	.6232	.5075	.2748	.5905	.5067	.2547	.6121	.4378	.1248
10	1e-4	24	1e-3	.5993	.5085	.2824	.5704	.4908	.2594	.6011	.4293	.1235
10	1e-4	24	5e-3	.5952	.5042	.2787	.5670	.4834	.2601	.5940	.4258	.1222
10	1e-4	24	1e-2	.5875	.4967	.2761	.5573	.4746	.2539	.5866	.4219	.1209
10	1e-4	32	1e-3	.5844	.4905	.2762	.5451	.4735	.2605	.5703	.4126	.1224
10	1e-4	32	5e-3	.5842	.4834	.2734	.5413	.4715	.2595	.5667	.4100	.1201
10	1e-4	32	1e-2	.5751	.4758	.2706	.5319	.4676	.2560	.5591	.4054	.1195
10	5e-4	16	1e-3	.6630	.5419	.3354	.6389	.5692	.2646	.7103	.4770	.1785
10	5e-4	16	5e-3	.6238	.5127	.3020	.6148	.5419	.2691	.6140	.4742	.1641
10	5e-4	16	1e-2	.5703	.4744	.3032	.5894	.4808	.2621	.5879	.4239	.1634
10	5e-4	24	1e-3	.6963	.6336	.3459	.7501	.6099	.3141	.7345	.5260	.1745
10	5e-4	24	5e-3	.6559	.5825	.3302	.7122	.5485	.2902	.6874	.4911	.1734
10	5e-4	24	1e-2	.6411	.5478	.2986	.6430	.5087	.2703	.6761	.4858	.1580
10	5e-4	32	1e-3	.7294	.6636	.3621	.7272	.5887	.3613	.7722	.5370	.1835
10	5e-4	32	5e-3	.6752	.6025	.3454	.6763	.5489	.3200	.7697	.5207	.1646
10	5e-4	32	1e-2	.6924	.5558	.3249	.6619	.5331	.3166	.7100	.4796	.1666
10	1e-3	16	1e-3	.5762	.5100	.2967	.5977	.5153	.2954	.5704	.4511	.1798
10	1e-3	16	5e-3	.5499	.4806	.2987	.5277	.4734	.2913	.5328	.4173	.1908
10	1e-3	16	1e-2	.5424	.4634	.3642	.5519	.4869	.3269	.5519	.4713	.3026
10	1e-3	24	1e-3	.6202	.5768	.3324	.6948	.5231	.3145	.6476	.5046	.1994
10	1e-3	24	5e-3	.5370	.4909	.3006	.5585	.4760	.2619	.5766	.4226	.1757
10	1e-3	24	1e-2	.5705	.4733	.3437	.5279	.4719	.3278	.5576	.4531	.2635
10	1e-3	32	1e-3	.6651	.5910	.3278	.6296	.5460	.3521	.7105	.4945	.1835
10	1e-3	32	5e-3	.5887	.5165	.3046	.5734	.4645	.3121	.6006	.4902	.1622
10	1e-3	32	1e-2	.5432	.4826	.3244	.5357	.4844	.3125	.5362	.4339	.2406
15	1e-4	16	1e-3	.7523	.6422	.3051	.7523	.6087	.2828	.7271	.5076	.1471
15	1e-4	16	5e-3	.7540	.6311	.3030	.7346	.6058	.2734	.7084	.4957	.1461
15	1e-4	16	1e-2	.7495	.6184	.2926	.7281	.5904	.2666	.7096	.4930	.1390
15	1e-4	24	1e-3	.7445	.6381	.3284	.6810	.5943	.2906	.7127	.5055	.1290
15	1e-4	24	5e-3	.7461	.6345	.3239	.6724	.5832	.2900	.6974	.4959	.1248
15	1e-4	24	1e-2	.7280	.6225	.3137	.6548	.5776	.2848	.6878	.4894	.1272
15	1e-4	32	1e-3	.7188	.6027	.3134	.6685	.5599	.2891	.6913	.4873	.1255
15	1e-4	32	5e-3	.7192	.5949	.3058	.6571	.5546	.2891	.6825	.4807	.1231
15	1e-4	32	1e-2	.7024	.5814	.2983	.6447	.5480	.2840	.6710	.4740	.1194
15	5e-4	16	1e-3	.7030	.6799	.3384	.7114	.6475	.3307	.7219	.5351	.1899
15	5e-4	16	5e-3	.6206	.5316	.3157	.6112	.4678	.2883	.6411	.4443	.1690
15	5e-4	16	1e-2	.5602	.4819	.2883	.5577	.4579	.2683	.5715	.4144	.1721
15	5e-4	24	1e-3	.7922	.7076	.3774	.8058	.6447	.3370	.8588	.5964	.1973
15	5e-4	24	5e-3	.7004	.6486	.3284	.6889	.5597	.3214	.7534	.4924	.1700
15	5e-4	24	1e-2	.6441	.5512	.2918	.6501	.5216	.2769	.6880	.4367	.1698

15	5e-4	32	1e-3	.8742	.6808	.4104	.8246	.6905	.3843	.8933	.6119	.1935
15	5e-4	32	5e-3	.7123	.6550	.3380	.6859	.6302	.3338	.7934	.5508	.1686
15	5e-4	32	1e-2	.6806	.6147	.3295	.6811	.5810	.3189	.7598	.5108	.1510
15	1e-3	16	1e-3	.6077	.5188	.3167	.5680	.4872	.3031	.6082	.4591	.2234
15	1e-3	16	5e-3	.5616	.4668	.3068	.5412	.4645	.3060	.5277	.4375	.2073
15	1e-3	16	1e-2	.5639	.5164	.3976	.5695	.4898	.3605	.5551	.4886	.3136
15	1e-3	24	1e-3	.6980	.5378	.3539	.6500	.5512	.3148	.7150	.5217	.2158
15	1e-3	24	5e-3	.6056	.4705	.3035	.5326	.4591	.2966	.5989	.4514	.2062
15	1e-3	24	1e-2	.5795	.4987	.3615	.5809	.4924	.3512	.5754	.4972	.2900
15	1e-3	32	1e-3	.7069	.5711	.3403	.6838	.5895	.3476	.7523	.5099	.1970
15	1e-3	32	5e-3	.5661	.4855	.3351	.5663	.4579	.2891	.6317	.4376	.2281
15	1e-3	32	1e-2	.5663	.4762	.3577	.5300	.4579	.3426	.6383	.4667	.3260

SUPPLEMENTARY TABLE 3. Hyperparmeter grid search results for Experiment 2. The 9 leftmost columns are the mean loss (the average binary cross entropy loss between the machine output and the crowd labels) averaged across the 30 training iterations for each of the training datasets in Experiment 2. The highlighted cells are the lowest mean loss for the entire column and therefore refer to the hyperparameter combination used for that dataset in the main text.
